# Yoda1‐Functionalized Nanozymes Coordinate Piezo1 Activation and Mitochondrial Protection to Reprogram the Diabetic Osteoimmune Micromilieu and Promote Vascularized Bone Repair

**DOI:** 10.1002/advs.78104

**Published:** 2026-10-14

**Authors:** Qing‐Song Deng, Ang Wang, Ren Zhang, Xu‐Ran Li, Po‐Lin Liu, Shu‐Hang He, Zi‐Yin Han, Zhi‐Han Shen, Zhan‐Ying Wei, Chang‐Ru Zhang, Fei Wang, Helen Dawes, Jian Lu, Tong‐He Zhu, Jin‐Zhong Zhao, Shang‐Chun Guo, Shi‐Cong Tao

**Affiliations:** ^1^ Department of Orthopaedic Surgery Shanghai Sixth People's Hospital Affiliated to Shanghai Jiao Tong University School of Medicine Shanghai China; ^2^ Department of Sports Medicine Shanghai Sixth People's Hospital Affiliated to Shanghai Jiao Tong University School of Medicine Shanghai China; ^3^ School of Medicine Shanghai Jiao Tong University Shanghai China; ^4^ Institute of Microsurgery on Extremities Shanghai Sixth People's Hospital Affiliated to Shanghai Jiao Tong University School of Medicine Shanghai China; ^5^ Jiangxi University of Chinese Medicine Nanchang China; ^6^ Shanghai Key Laboratory of Orthopaedic Implants Department of Orthopaedic Surgery Shanghai Ninth People's Hospital Shanghai Jiao Tong University School of Medicine Shanghai China; ^7^ Leicester Medical School University of Leicester Leicester UK; ^8^ Shanghai Clinical Research Centre of Bone Diseases Department of Osteoporosis and Bone Diseases Shanghai Sixth People's Hospital Affiliated to Shanghai Jiao Tong University School of Medicine Shanghai China; ^9^ Clinical and Translational Research Centre for 3D Printing Technology Medical 3D Printing Innovation Research Centre Shanghai Ninth People's Hospital Shanghai Jiao Tong University School of Medicine Shanghai China; ^10^ Department of Orthopaedics, Shanghai Key Laboratory for Prevention and Treatment of Bone and Joint Diseases Shanghai Institute of Traumatology and Orthopaedics Ruijin Hospital Shanghai Jiao Tong University School of Medicine Shanghai China; ^11^ Faculty of Health and Life Science Oxford Brookes University Oxford UK; ^12^ NIHR Oxford Health Biomedical Research Centre Oxford UK; ^13^ College of Medicine and Health St Lukes Campus University of Exeter Exeter UK; ^14^ Department of Orthopaedic Surgery Shanghai Eighth People's Hospital Shanghai China; ^15^ Multidisciplinary Innovation Centre for Bioadditive Manufacturing Institute for Frontier Medical Technology School of Chemistry and Chemical Engineering Shanghai University of Engineering Science Shanghai China

**Keywords:** 3D‐printed diamond lattice scaffold, mitochondrial homeostasis, nanozyme, vascularized bone repair, Yoda1

## Abstract

Despite advances in bone tissue engineering, repairing diabetic bone defects remains a formidable challenge. Here, we present an innovative strategy for diabetic bone regeneration, the BCPG‐MF@Y scaffold, which features diamond lattices of 3D‐printed biphasic calcium phosphate (BCP) integrating porous gelatine methacryloyl (pGelMA) hydrogels embedded with Yoda1‐functionalized manganese dioxide‐ferritin (MF@Y) biomimetic nanozymes. Our comprehensive analyses revealed that the MF@Y nanozyme orchestrates mitochondrial homeostasis in bone mesenchymal stem cells (BMSCs), effectively scavenges mtROS, maintains mitochondrial membrane potential, modulates mitochondrial permeability transition pore dynamics, and prevents mitochondrial DNA leakage into the cytosol. This leads to inhibition of cGAS‒STING pathway activation, rejuvenation of senescent cells, reduced inflammation, and control of BMSC differentiation. Yoda1‐mediated activation of Piezo1 mimics mechanical stimulation, promoting osteogenic differentiation while inhibiting adipogenic and osteoclastic differentiation. The BCPG‐MF@Y scaffold synergistically reprograms the osteoimmune micromilieu by protecting mitochondria and activating Piezo1, showing significant promise for vascularized bone repair in diabetic conditions and potential applications in chronic inflammatory disorders.

## Graphical abstract

Diabetic bone defects remain a critical clinical challenge. A 3D‐printed BCPG‐MF@Y scaffold, embedding Yoda1‐functionalized manganese dioxide‐ferritin nanozymes, scavenges mtROS, suppresses the cGAS–STING pathway, and reverses cellular senescence, while Yoda1 activates Piezo1 to direct BMSCs fate to synergistically reprogram the osteoimmune microenvironment for vascularized bone regeneration under hyperglycemic conditions.

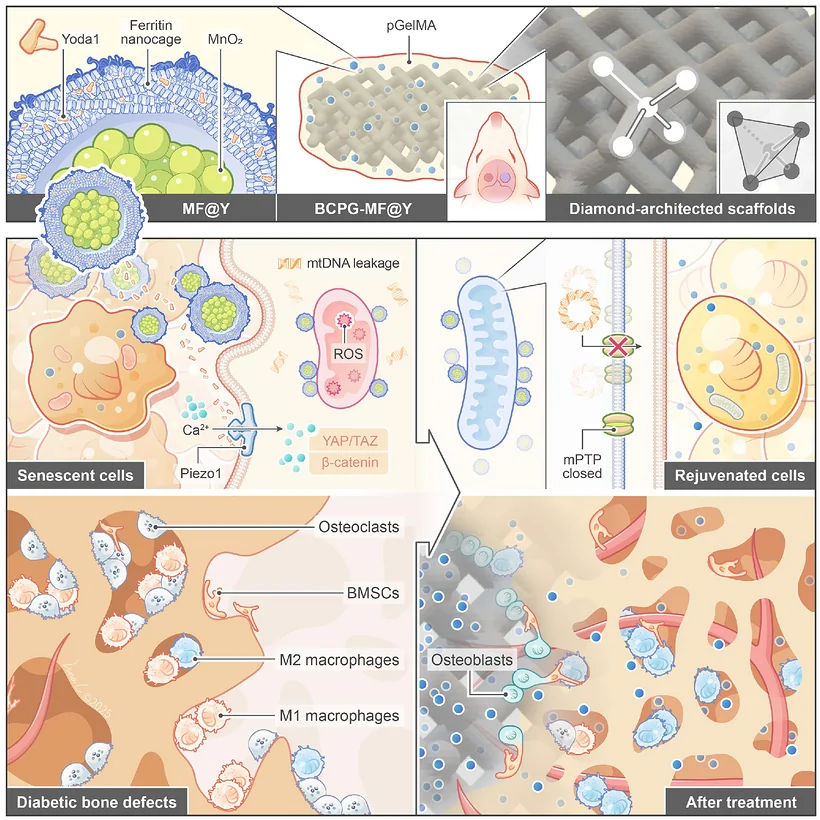

## 1 Introduction

Diabetes mellitus has emerged as a formidable global health challenge, affecting over 537 million adults (10.5% of the world's population), and is characterized by chronic metabolic dysregulation that manifests as persistent hyperglycemia [1, 2, 3, 4]. While conventional methods for diabetes management focus on glycaemic control, the complex skeletal complications associated with this disease have been uncovered due to its increasing prevalence [5]. Of particular concern are bone defects, which are associated with a 1.79‐fold greater fracture risk and threefold greater nonunion rate in diabetic patients than in individuals without diabetes [6, 7, 8]. Unlike postmenopausal osteoporosis or mechanical insufficiency‐induced nonunion, diabetic bone defects are aggravated through a self‐perpetuating metabolic‒osteoimmune axis [9, 10, 11, 12]. Sustained hyperglycemia initiates mitochondrial dysfunction‐mediated overproduction of reactive oxygen species (ROS), which synergizes with cellular senescence and chronic inflammation [13, 14, 15]. This cascade uniquely orchestrates the dual pathology of the suppression of osteogenic signaling and acceleration of bone resorption, a mechanism distinct from those of glucocorticoid‐induced or age‐related bone loss [10, 16]. This multifaceted pathophysiology, distinct from those of other bone disorders, necessitates mechanism‐specific strategies that target the oxidative stress–senescence–inflammation feedback loop.

While the precise mechanisms driving diabetic bone defects have yet to be fully elucidated, excessive ROS accumulation caused by the dysregulation of mitochondrial homeostasis has emerged as a critical pathogenic determinant, as it initiates a series of downstream pathological cascades [13, 14]. For example, in patients with diabetes, persistent hyperglycemia results in dysregulated mitochondrial homeostasis, which impairs mitochondrial electron transport chain (ETC) function and leads to significant ROS generation, further exacerbating mitochondrial dysfunction [17]. In addition, the accumulation of mitochondrial ROS (mtROS) leads to mitochondrial protein oxidation and mitochondrial DNA (mtDNA) damage and can induce the opening of the mitochondrial permeability transition pore (mPTP), facilitating the release of mtDNA into the cytoplasm, which activates the cyclic guanosine monophosphate—adenosine monophosphate synthetase—stimulator of interferon genes (cGAS‒STING) pathway, promoting cellular senescence and the secretion of inflammatory factors [18, 19]. Notably, the cGAS–STING pathway is increasingly recognized as a pivotal mediator of diabetic bone defects. Within the diabetic bone microenvironment, chronic activation of the cGAS–STING pathway in osteoblasts suppresses osteogenic differentiation through downregulation of key transcription factors, including RUNX2 and Osterix, while concurrently promoting osteoblast apoptosis via mitochondria‐dependent pathways [20]. Furthermore, STING activation in bone mesenchymal stem cells (BMSCs) compromises both their proliferative capacity and osteogenic potential, thereby impairing bone formation [21]. The cGAS–STING pathway also exacerbates osteoclastogenesis by enhancing RANKL expression in osteocytes, creating an imbalance in bone remodeling that favors resorption over formation [22, 23]. Moreover, excessive production of ROS influences macrophage polarization by promoting M1 macrophage polarization and inhibiting M2 macrophage polarization, which also leads to chronic inflammation and impedes tissue regeneration [24, 25]. In addition, in the presence of large amounts of ROS, excessive osteoclast differentiation occurs, accelerating bone loss [26].

Chronic hyperglycemia in diabetic patients drives mitochondrial dysfunction and increases oxidative stress, inducing cellular senescence that critically compromises healing of bone defects owing to the functional impairment of mesenchymal stem cells and endothelial cells during bone regeneration [27]. Senescent BMSCs exhibit a significantly decreased capacity for osteogenic differentiation and a pronounced tendency towards adipogenic differentiation, which impedes bone regeneration [28, 29]. Furthermore, senescent endothelial cells exhibit markedly diminished angiogenic potential, thereby impairing neovascularization and failing to deliver the oxygen, nutrients, and osteogenic growth factors critical for bone regeneration [30]. Moreover, senescent cells secrete various senescence‐associated secretory phenotype (SASP) factors and proinflammatory cytokines, which further aggravate cellular senescence [31]. These factors collectively disrupt bone metabolism, leading to decreased bone formation and increased bone resorption, posing extreme challenges to bone regeneration in diabetic bone defects.

Given that this metabolic imbalance is orchestrated by mitochondrial dysfunction and cellular senescence, recent research has highlighted mechanical stimulation as a potent modulator capable of restoring bone homeostasis [32]. Recently, the involvement of the mechanosensitive Piezo1 channel in bone metabolism has garnered increasing attention. Piezo1 functions as a mechanosensitive Ca^2+^‐permeable nonselective cation channel that transduces a variety of mechanical cues into intracellular Ca^2+^ signals [33]. This mechanoelectrical transduction then activates calcineurin‐dependent signaling pathways to regulate bone homeostasis [34]. In BMSCs, Piezo1 activation significantly promotes osteogenic differentiation while suppressing adipogenic differentiation [35, 36]. Moreover, the activation of Piezo1 in osteoblasts and osteocytes has been shown to suppress osteoclast‐mediated bone resorption [32]. Additionally, activation of Piezo1 expressed in the membranes of vascular endothelial cells facilitates angiogenesis [37]. Notably, Piezo1 also plays a crucial role in mitigating age‐associated bone loss [38]. However, accumulating evidence points to a critical interplay between oxidative stress and Piezo1 function in diabetic bone defects. ROS accumulation in hyperglycaemic environments suppresses Piezo1 expression and activity [35, 39]. In turn, Piezo1 dysfunction further compromises mechanosensitivity and impairs the activation of osteogenic transcription factors, including RUNX2 and Osterix [40], thereby establishing a pathological feed‐forward cycle that perpetuates impaired bone formation. In support of this mechanism, using osteoblast‐specific Piezo1 knockout mice, Wang et al. demonstrated that Piezo1 deficiency leads to pronounced bone loss, reduced bone mineral density, and impaired bone defect healing [32]. Complementing these findings, Zhou et al. revealed that Piezo1/2‐mediated mechanotransduction drives bone formation through coordinated activation of the NFAT–YAP1–β‐catenin signaling axis [35], further underscoring the indispensable role of Piezo1 in maintaining skeletal homeostasis. Taken together, these observations delineate a pathological cascade in diabetic bone disease in which hyperglycemia drives ROS accumulation, triggering mitochondrial DNA damage, cGAS–STING activation, and a sustained inflammatory response that collectively suppress Piezo1 expression and ultimately impair bone formation.

Thus, there has been increasing interest in exploiting this mechanobiological axis for therapeutic purposes, and pharmacologically activating Piezo1 has emerged as a strategic intervention. In this context, the low‐molecular‐weight compound Yoda1 represents a first‐in‐class chemical agonist capable of activating Piezo1 channels independently of mechanical stimulation [41]. However, the recalcitrant nature of diabetic bone defects fundamentally arises from the multifactorial pathophysiology that disrupts bone metabolic homeostasis [42, 43]. This pathological complexity leads to the establishment of a self‐perpetuating microenvironment, which intrinsically compromises the efficacy of Piezo1‐mediated mechanotransduction [44]. Therefore, it is imperative to implement a combinatorial therapeutic strategy integrating Yoda1 with anti‐senescent agents, ROS scavengers, and anti‐inflammatory molecules to reset the pathological microenvironment and restore mechanobiological signaling simultaneously.

In this study, we aimed to determine whether 3D‐printed diamond cubic lattice biphasic calcium phosphate (BCP) scaffolds integrating porous gelatine methacryloyl (pGelMA) hydrogels embedded with Yoda1‐functionalized manganese dioxide‐ferritin (MF@Y) bioactive nanozymes could promote the healing of diabetic bone defects. To address this question, we first generated the MF@Y nanozyme through molecular self‐assembly and fabricated the BCP scaffold via precision digital light processing (DLP) 3D printing before combining these components to construct the BCPG‐MF@Y composite scaffold. Mechanistically, RNA‐seq analysis revealed that the MF@Y nanozyme orchestrates the modulation of multiple pathways, specifically targeting those related to diabetic pathophysiology, cellular senescence, immunometabolic reprogramming, bone remodeling processes, and mitochondrial function. We then performed in vitro functional studies and in vivo experiments with diabetic bone defect models. Consistent with the mechanistic insights, microcomputed tomography (micro‐CT) and histological analyses revealed that the BCPG‐MF@Y scaffold significantly enhanced bone regeneration. Notably, the therapeutic efficacy of the BCPG‐MF@Y system stems from the pioneering osteoimmune microenvironment‐reprogramming strategy, which synergistically operates through multiple mechanisms, including the establishment of a pro‐osteogenic niche via Yoda1‐mediated Piezo1 activation, the neutralization of oxidative‐inflammatory stress through MF@Y nanozyme‐mediated ROS scavenging, and the reversal of cellular senescence by maintaining mitochondrial homeostasis. Collectively, this multifunctional synergistic biomaterial strategy is the first to simultaneously reprogram the diabetic bone microenvironment and restore mechanotransduction–osteogenesis coupling, thereby establishing the BCPG‐MF@Y scaffold as a transformative therapeutic platform for diabetic bone regeneration.

## 2 Results

### 2.1 Characterization of the MF Nanozyme

To investigate the therapeutic potential of the BCPG‐MF@Y scaffold in the healing of diabetic bone defects, the MF nanozyme was developed using recombinant human ferritin heavy chain (HFn) as a structural foundation. HFn, considered an ideal nanoscale scaffold, offers distinct advantages for nanozyme development due to its hollow nanocage architecture, a structural feature that facilitates the precise encapsulation of metal oxide nanoparticles while preserving their catalytic enzyme‐mimicking properties [45, 46]. Building upon this structural framework, the MF nanozyme was developed through the in situ incorporation of manganese dioxide (MnO_2_) within the engineered HFn nanocages. Initially, transmission electron microscopy (TEM) and energy‐dispersive X‐ray spectroscopy (EDS) were employed to investigate the morphology and elemental composition of the nanozymes, respectively. TEM revealed numerous nanoparticles embedded within the MF nanozyme core (Figure 1A and Figure S1), and EDS analysis confirmed the high manganese content within these particles (Figure 1B,C). Subsequently, Fourier transform infrared (FTIR) spectroscopy was used to examine the chemical bonds within the MF nanozymes. Notably, a characteristic absorption peak at 670 cm^−1^ was observed, indicating the presence of Mn‐O bonds (Figure 1D). Furthermore, electron paramagnetic resonance (EPR) was employed to evaluate the catalytic functions of the MF nanozyme. The results revealed a significant decrease in the levels of both O_2_^•−^ and •OH radicals with increasing MF nanozyme concentration (Figure 1E,F), suggesting the ability of the nanozyme to scavenge these ROS effectively. Finally, dynamic light scattering (DLS) was used to determine the size distribution of the MF nanozyme, which showed a mean diameter of 98.99 ± 4.93 nm (Figure 1G). These findings indicate the successful synthesis of monodisperse MF nanozymes that exhibit potent O_2_^•−^ and •OH radical scavenging activity.

**FIGURE 1:**
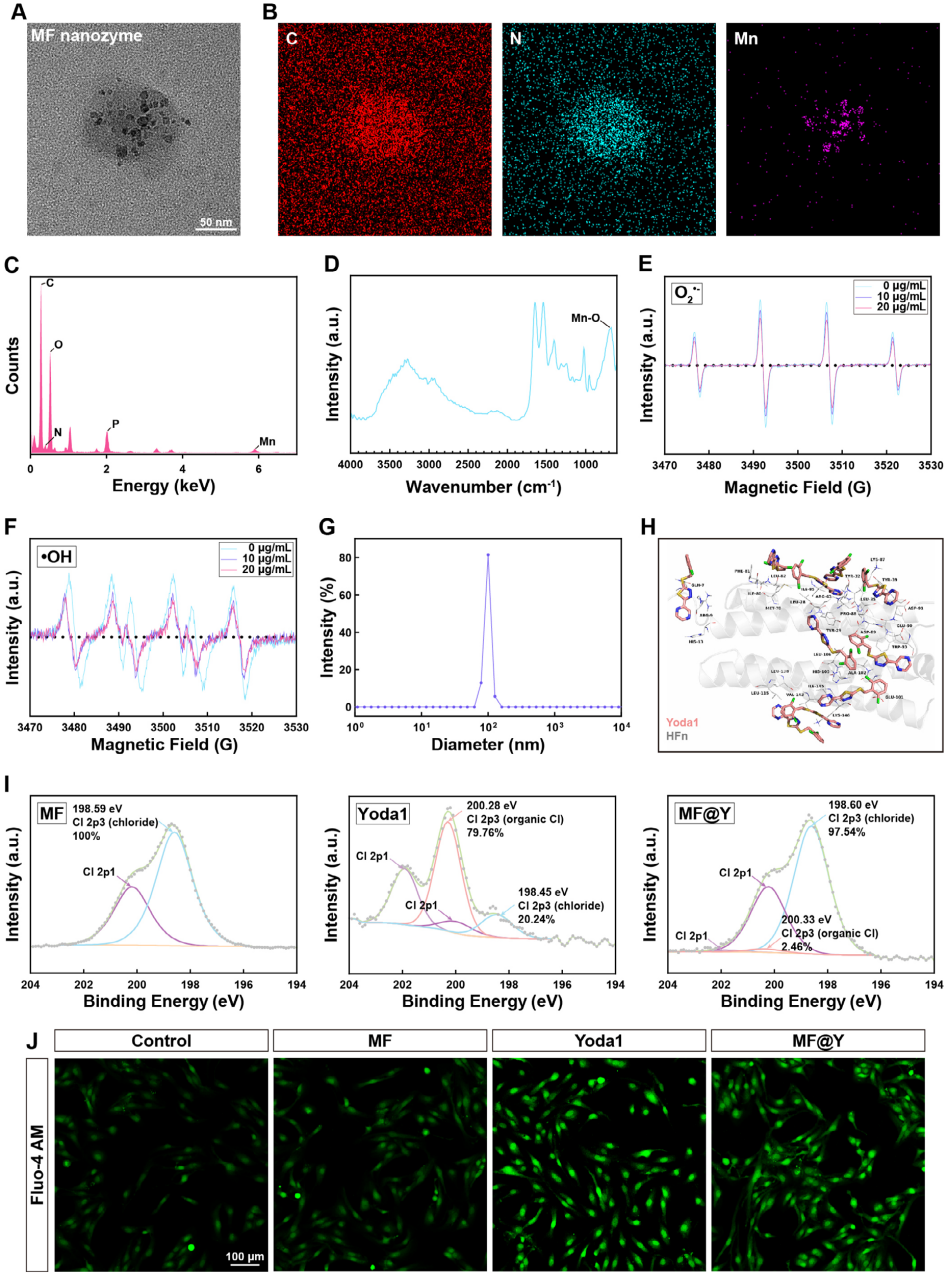
Characteristics of the MF and MF@Y nanozymes. (A) Transmission electron microscopy (TEM) images of the MF nanozyme. (B) Elemental mapping of C, N, and Mn in the MF nanozyme. (C) Energy‐dispersive X‐ray spectroscopy (EDS) spectrum of the MF nanozyme. (D) Fourier transform infrared (FTIR) spectrum of the MF nanozyme. The enzyme‐like activities of the MF nanozyme in scavenging O_2_^−−^ (E) and •OH (F) evaluated via electron paramagnetic resonance (EPR). (G) Particle size distribution of the MF nanozymes. (H) Molecular dynamics simulations of a subunit of HFn and Yoda1. (I) XPS Cl spectra of the MF nanozyme, Yoda1, and the MF@Y nanozyme. (J) Fluo‐4 AM staining to evaluate the calcium ion concentration.

### 2.2 Formulation and Characterization of the MF@Y Nanozyme

The MF nanozyme, composed of a cage‐like recombinant HFn scaffold encapsulating a core of MnO_2_ nanoparticles, possesses abundant hydrophobic regions within its protein architecture [47, 48]. Given the hydrophobic nature of Yoda1, these structural features motivated us to investigate the binding mechanism of Yoda1 to the MF nanozyme through molecular docking and molecular dynamics simulations. Considering that HFn consists of 12 identical subunits, we selected a single subunit of the protein cage to perform the simulations to optimize computational efficiency while maintaining the structural features essential for Yoda1 binding. As shown in Figure 1H, 10 Yoda1 molecules adopted compact conformations upon binding to the pockets of HFn. These Yoda1 molecules were located at the hydrophobic site surrounded by the residues Leu‐28, Leu‐35, Met‐70, Ile‐80, Phe‐81, Leu‐82, Ile‐85, Pro‐88, Trp‐93, Ala‐102, Leu‐106, Leu‐115, Leu‐138, Val‐142, and Ile‐145, resulting in strong hydrophobic binding interactions. Detailed analysis revealed that anion‒π interactions formed between Yoda1 and residues Asp‐89, Glu‐90, Asp‐91, and Glu‐101 of HFn; π‒π stacking interactions formed between one Yoda1 molecule and residues Tyr‐29 and Trp‐93; and cation‒π interactions formed between Yoda1 and residues Arg‐9, Arg‐63, Lys‐87, and Lys‐146 of HFn. These results indicated that Yoda1 can be loaded onto the MF nanozyme.

After determining that Yoda1 could theoretically be loaded onto the MF nanozyme, we next sought to confirm the formation of the Yoda1‐functionalized MF (MF@Y) nanozyme in vitro via X‐ray photoelectron spectroscopy (XPS). As shown in Figure 1I, the MF nanozyme spectrum exhibited a Cl 2p3 peak at 198.59 eV, which corresponds to chloride. In the Yoda1 spectrum, there were two distinct Cl 2p3 peaks at 198.45 eV and 200.28 eV, indicating the presence of inorganic chloride and organic chlorine, respectively. Similarly, in the MF@Y nanozyme spectrum, two Cl 2p3 peaks were present at 198.60 eV and 200.33 eV, representing inorganic chloride and organic chlorine, respectively. These data indicated that organic chlorine serves as a distinctive marker of Yoda1 incorporated into the MF@Y nanozyme and confirmed the adsorption of Yoda1.

Moreover, we employed Fluo‐4 AM staining to evaluate the calcium ion concentration in cells following treatment with the MF@Y nanozyme to assess the activity of Yoda1 after its incorporation, as Yoda1 is a stimulator of the calcium ion channel Piezo1. As shown in Figure 1J, both the control and MF groups presented weak green fluorescence, whereas the Yoda1 and MF@Y groups presented markedly stronger green fluorescence. These results indicated that Yoda1 and the MF@Y nanozyme (due to the incorporation of Yoda1), but not the MF nanozyme, can increase the intracellular calcium ion concentration.

### 2.3 Bioinformatic Analysis of the Biological Effects of the MF@Y Nanozyme

Following successful development of the MF@Y nanozyme specifically engineered to target the pathological mechanism of diabetic bone defects, we systematically investigated its biological functions. First, RNA‐seq was employed to assess changes in gene transcription following treatment of human BMSCs (HBMSCs) with the MF@Y nanozyme. As depicted in Figure 2A,B, 407 significantly differentially expressed genes (DEGs) were identified between the MF@Y nanozyme (treated) and control (untreated) groups, of which 176 were upregulated and 231 were downregulated. Pathway enrichment analysis of the DEGs was subsequently conducted using the KEGG, GO, Reactome, and WikiPathways databases. As shown in Figure 2C and Figure S2, diabetes‐related pathways (type I diabetes mellitus and AGE‐RAGE signaling pathway in diabetic complications), senescence‐related pathways (AMPK signaling pathway, and senescence‐associated secretory phenotype (SASP)), immune‐related pathways (interferon alpha/beta signaling, response to type I interferon and negative regulation of NF‐κB transcription factor activity), osteogenesis‐related pathways (canonical Wnt signaling pathway, BMP receptor binding and positive regulation of osteoblast differentiation), angiogenesis‐related pathways (positive regulation of angiogenesis and signaling by PDGF), osteoclastogenesis‐related pathways (negative regulation of osteoclast differentiation and osteoclast signaling), and adipogenic differentiation‐related pathways (adipogenesis and fat cell differentiation) were significantly enriched in the DEGs. Furthermore, gene set enrichment analysis (GSEA) was performed on all the genes, and the results showed the significant activation of numerous mitochondria‐associated processes, including protein import into the mitochondrial matrix, superoxide metabolic processes, energy homeostasis, and the response to oxidative stress, after MF@Y nanozyme treatment (Figure 2D). In conclusion, RNA‐seq analysis indicated that the MF@Y nanozyme regulates key pathways involved in diabetes, senescence, immunity, osteogenesis, angiogenesis, adipogenesis, and mitochondrial function, which suggests that the MF@Y nanozyme is a potential therapeutic agent for treating diabetic bone defects.

**FIGURE 2:**
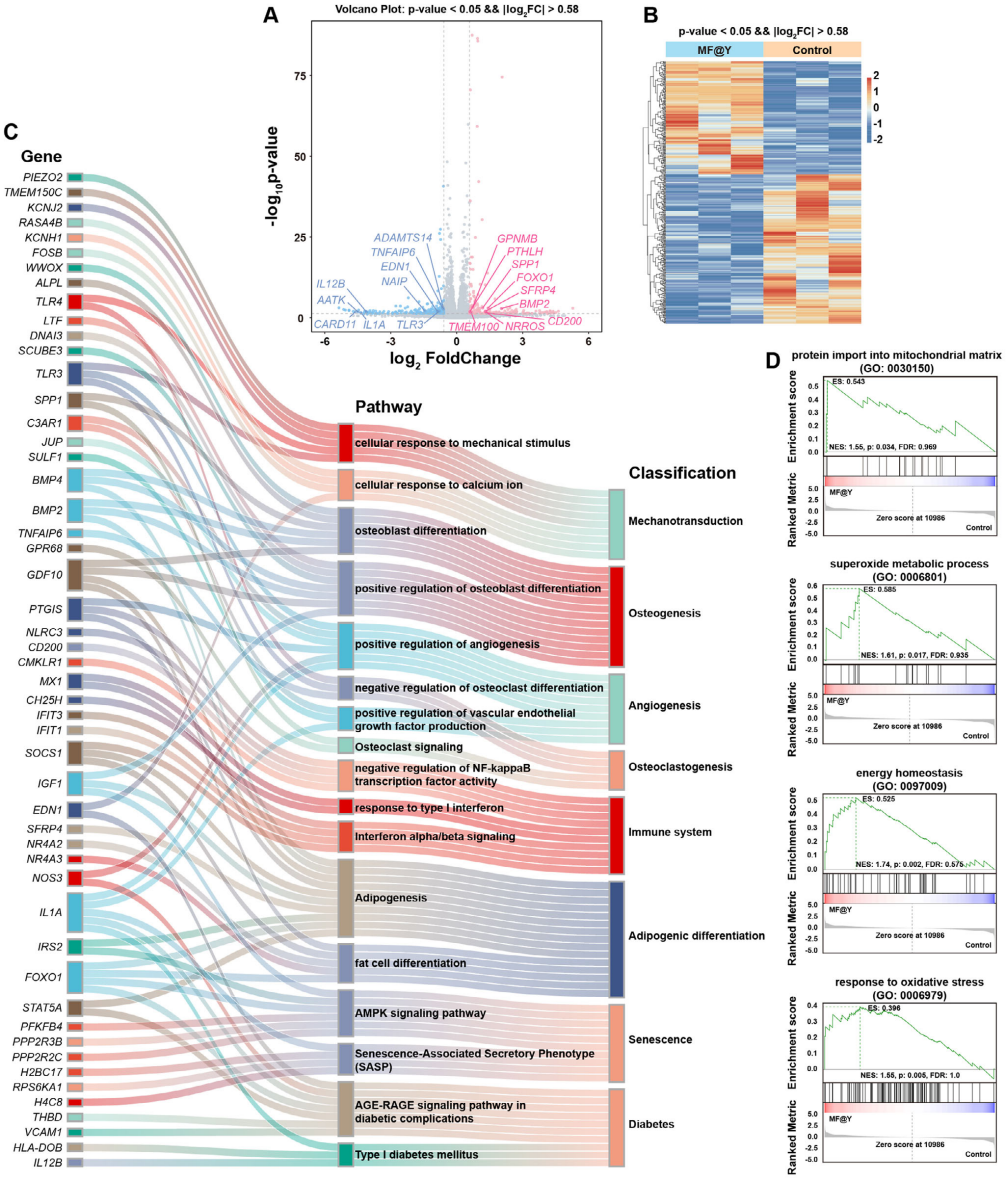
Exploration of the biological effects of the MF@Y nanozyme using RNA‐seq. (A) Volcano plots of the differentially expressed genes (DEGs) after MF@Y nanozyme treatment. (B) Cluster analysis of the DEGs. (C) Sankey analysis diagram of the enriched GO terms, WikiPathways, Reactome, and KEGG pathways. (D) The results of gene set enrichment analysis (GSEA).

### 2.4 The Dynamic Transport Pathway of the MF@Y Nanozyme

To delineate the intracellular trafficking route of the MF@Y nanozyme, we fluorescently labeled the nanozyme and used antibodies against established organelle markers, Rab5 (early endosomes), TOM20 (mitochondria) and LAMP1 (lysosomes), to monitor organelle‐specific colocalization at defined time points by confocal microscopy. As shown in Figure S3A,B, robust colocalization with early endosomes was evident within 30 min of nanoparticle addition, consistent with internalization via the endocytic pathway and initial trafficking into the early endosomal compartment. Colocalization with early endosomes subsequently declined and reached a plateau, indicative of endosomal escape and onward redistribution to downstream organelles. Time‐resolved imaging further revealed that internalized nanoparticles engage two parallel trafficking routes, the mitochondrial route and the lysosomal route. The proportion of nanoparticles colocalizing with mitochondria increased continuously throughout the observation period, identifying mitochondria as the predominant terminal destination. By contrast, lysosomal colocalization exhibited a biphasic profile, rising to a peak at early time points before declining markedly at later ones. This transient lysosomal accumulation, temporally offset from the sustained rise in mitochondrial colocalization, suggests that a population of nanoparticles undergoes brief lysosomal sequestration prior to lysosomal escape and ultimate mitochondrial enrichment. Collectively, these data define a stepwise intracellular trafficking program for MF@Y nanozymes: endocytic capture into early endosomes, transient lysosomal retention, lysosomal escape, and preferential mitochondrial accumulation. These findings establish the mitochondria‐targeting competence of MF@Y nanozymes and implicate mitochondria as the primary subcellular site at which the nanozymes exert their biological activity.

### 2.5 The MF@Y Nanozyme Alleviates Oxidation‐Induced ROS Accumulation and Changes in Mitochondrial Morphology

Building upon the pathway regulation capabilities of the MF@Y nanozyme identified through RNA‐seq analysis, we specifically investigated the antioxidative potential of the nanozyme against ROS‐dominated pathological microenvironments–a critical barrier in diabetic bone regeneration. The accumulation of large amounts of ROS at the diabetic bone defect site is one of the most significant factors leading to a lack of healing, as elevated ROS levels can induce cellular dysfunction and senescence [49]. To mimic the high ROS levels in the diabetic bone defect microenvironment, tert‐butyl hydroperoxide (TBHP), a stable and persistent oxidizer [50, 51], was added to the HBMSCs culture medium. First, 2’,7’‐dichlorofluorescein diacetate (DCFH‐DA) staining was employed to evaluate the ROS‐scavenging properties of the designed nanozyme. As shown in Figure S4A, under normal conditions without TBHP treatment (control group and MF@Y group), few cells exhibited green fluorescence. In contrast, after treatment with TBHP, many cells displayed green fluorescence, indicating the substantial accumulation of intracellular ROS. Notably, the addition of the MF@Y nanozyme to the high ROS cell culture medium resulted in a marked reduction in intracellular ROS levels. These data indicated the excellent antioxidative and ROS‐scavenging properties of the MF@Y nanozyme. Given that mitochondria constitute the primary source of cellular ROS, we next assessed the effects of the MF@Y nanozyme on mitochondrial ROS (mtROS) using the specific indicator probe MitoSOX Green. As shown in Figure 3A and Figure S4B, substantial amounts of mtROS were generated following treatment with TBHP. However, upon treatment with the MF@Y nanozyme, the green fluorescence intensity within the mitochondria dramatically decreased. These data indicated that TBHP increases mtROS production, whereas the MF@Y nanozyme effectively reduces mtROS accumulation.

**FIGURE 3:**
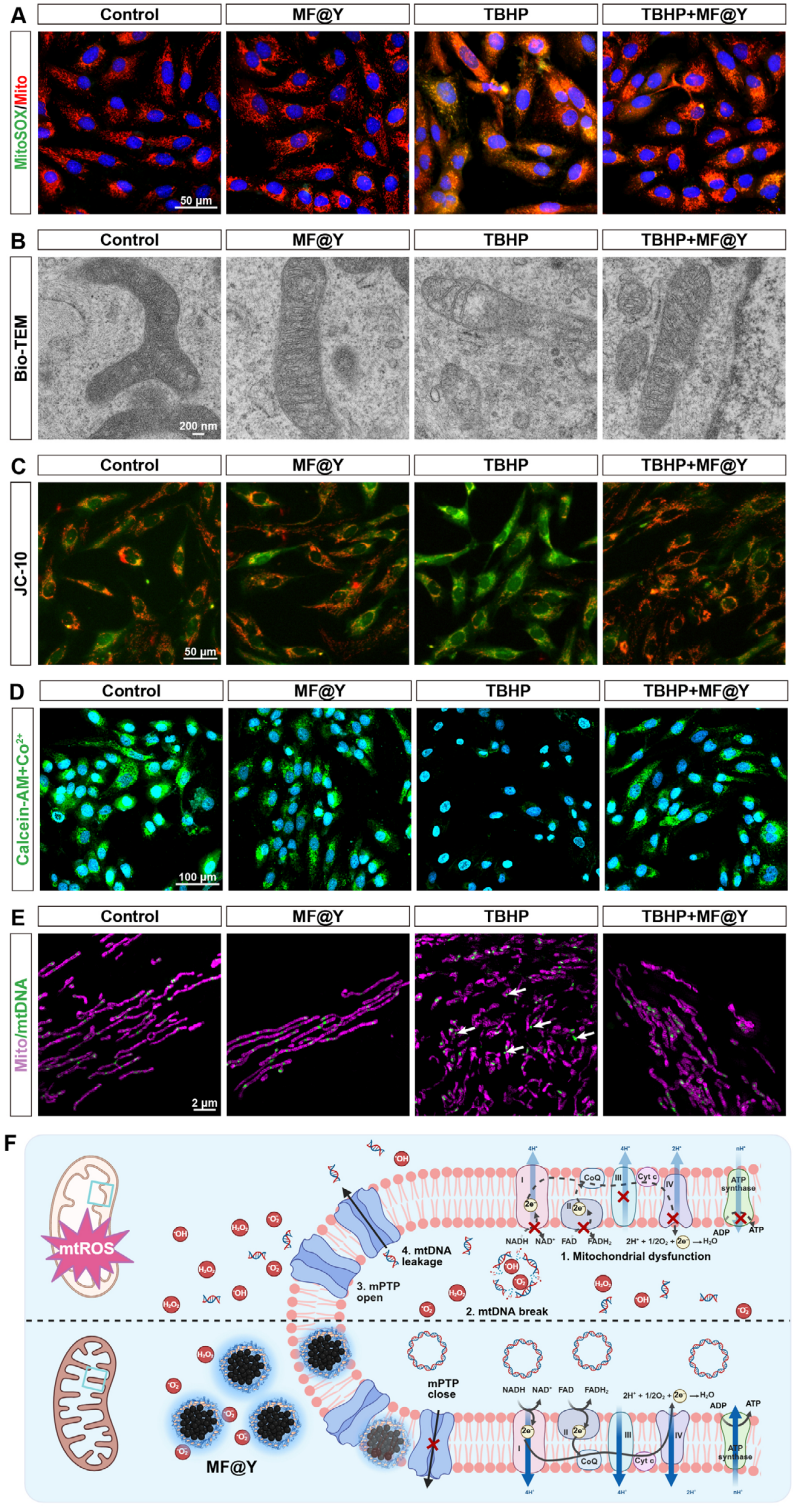
Effects of the MF@Y nanozyme on antioxidant and mitochondrial homeostasis. (A) Fluorescence images of MitoSOX Green‐stained mtROS in HBMSCs. (B) Bio‐TEM images showing the morphology and structure of the mitochondria. (C) Fluorescence images of JC‐10 to evaluate mitochondrial membrane potential. (D) Fluorescence images of calcein‐AM staining after treatment with CoCl_2_ to evaluate opening of the mPTP. (E) Fluorescence images of mitochondria and mtDNA to asses mtDNA translocation into the cytosol. (F) Schematic diagram showing the effects of MF@Y nanozyme on mitochondrial homeostasis. This illustration was created with BioRender.com.

Furthermore, bio‐TEM was employed to observe morphological and structural alterations in the cells to further evaluate the ability of the MF@Y nanozyme to alleviate the impact of high ROS levels. As shown in Figure 3B, following exposure to TBHP, notable changes in mitochondrial morphology were observed. Compared with those in the control group, the mitochondria in the TBHP‐treated group were swollen, and this change was accompanied by the disappearance or vacuolation of the mitochondrial cristae. In contrast, the structural integrity and cristae organization of the mitochondria in the TBHP+MF@Y group were maintained. These results suggest that the MF@Y nanozyme may act on mitochondria to alleviate damage caused by ROS and maintain mitochondrial homeostasis (Figure S4C).

### 2.6 The MF@Y Nanozyme Modulates Mitochondrial Homeostasis

To further validate the effects of the MF@Y nanozyme on mitochondrial function, JC‐10 staining was next employed to assess changes in the mitochondrial membrane potential (ΔΨm) in response to MF@Y nanozyme treatment. A high ΔΨm promotes the formation of JC‐10 aggregates in mitochondria, which emit red fluorescence, whereas depolarization triggers the dispersion of JC‐10 monomers into the cytoplasm, which emit green fluorescence. As shown in Figure 3C and Figure S4D, TBHP‐induced oxidative stress substantially decreased the ΔΨm, resulting in increased green fluorescence intensity relative to that of controls. Compared with that in the TBHP group, the ΔΨm in the TBHP+MF@Y group significantly increased, and many cells exhibited red fluorescence. These results indicate that the MF@Y nanozyme effectively counteracts mitochondrial dysfunction by significantly increasing the ΔΨm, even in the presence of high levels of ROS. To further assess the functional consequences for cellular bioenergetics, we measured ATP production in the presence of MF@Y nanozymes. As shown in Figure S4E, MF@Y nanozyme treatment markedly rescued the reduction in ATP generation induced by TBHP, indicating that restoration of ΔΨm translates into recovery of oxidative phosphorylation capacity.

Next, we assessed the opening of the mPTP upon the addition of the MF@Y nanozyme to the cell medium in the presence of high levels of ROS. The results revealed that high ROS levels significantly increased the opening of the mPTP, whereas the MF@Y nanozyme reduced mPTP opening (Figure 3D and Figure S4F). These results indicate that the MF@Y nanozyme mitigates the excessive opening of the mPTP caused by increased mtROS levels. Previous investigations have shown that excessive mtROS production induces pathological activation of the mPTP, triggering mtDNA efflux into the cytosol [52, 53]. Building upon the results of the mPTP assessments, we examined the distribution of mtDNA. As shown in Figure 3E, under normal conditions (control), the mitochondria generally exhibited an elongated rod‐like morphology, and mtDNA was predominantly localized within these organelles. However, in the TBHP group, the mitochondria were obviously smaller and swollen, and mtDNA was present not only within the mitochondria but also to a significant extent in the cytoplasm. However, in the TBHP+MF@Y group, the elongated rod‐like mitochondrial morphology was maintained, and mtDNA was present predominantly within the mitochondria. These results indicate that the MF@Y nanozyme orchestrates mitochondrial homeostasis by maintaining mitochondrial membrane potential, modulating permeability transition pore dynamics, and preventing mitochondrial DNA breakage and leakage into the cytosol (Figure 3F).

### 2.7 The MF@Y Nanozyme Blocks cGAS‒STING Signaling and Alleviates Cellular Senescence

Many studies have suggested that in the presence of high levels of ROS, mtDNA is released into the cytoplasm through the mPTP, subsequently activating the cGAS‒STING pathway [53, 54]. Consequently, we further evaluated STING phosphorylation via immunofluorescence staining of phosphorylated STING (p‐STING). As shown in Figure 4A and Figure S5A, compared with that in the control group, expression of p‐STING in the TBHP group was significantly higher, whereas addition of the MF@Y nanozyme to the cell culture medium reversed this change, indicating that the MF@Y nanozyme inhibited cGAS‒STING pathway activation. Additionally, real‐time quantitative polymerase chain reaction (RT‐qPCR) was employed to measure the expression of interferon‐related genes (*IFNA1*, *IFNB1*, and *IFNA2*), and SASP genes (interleukin‐6 (*IL‐6*), C‐X‐C motif chemokine ligand 1 (*CXCL1*), *CXCL10*, and matrix metalloproteinase 1 (*MMP1*)). Compared with the TBHP group, the TBHP+MF@Y group expressed significantly lower levels of these genes (Figure 4B and Figure S5B), indicating that the MF@Y nanozyme inhibits activation of the cGAS‒STING pathway and its downstream signaling pathways (Figure S5C).

**FIGURE 4:**
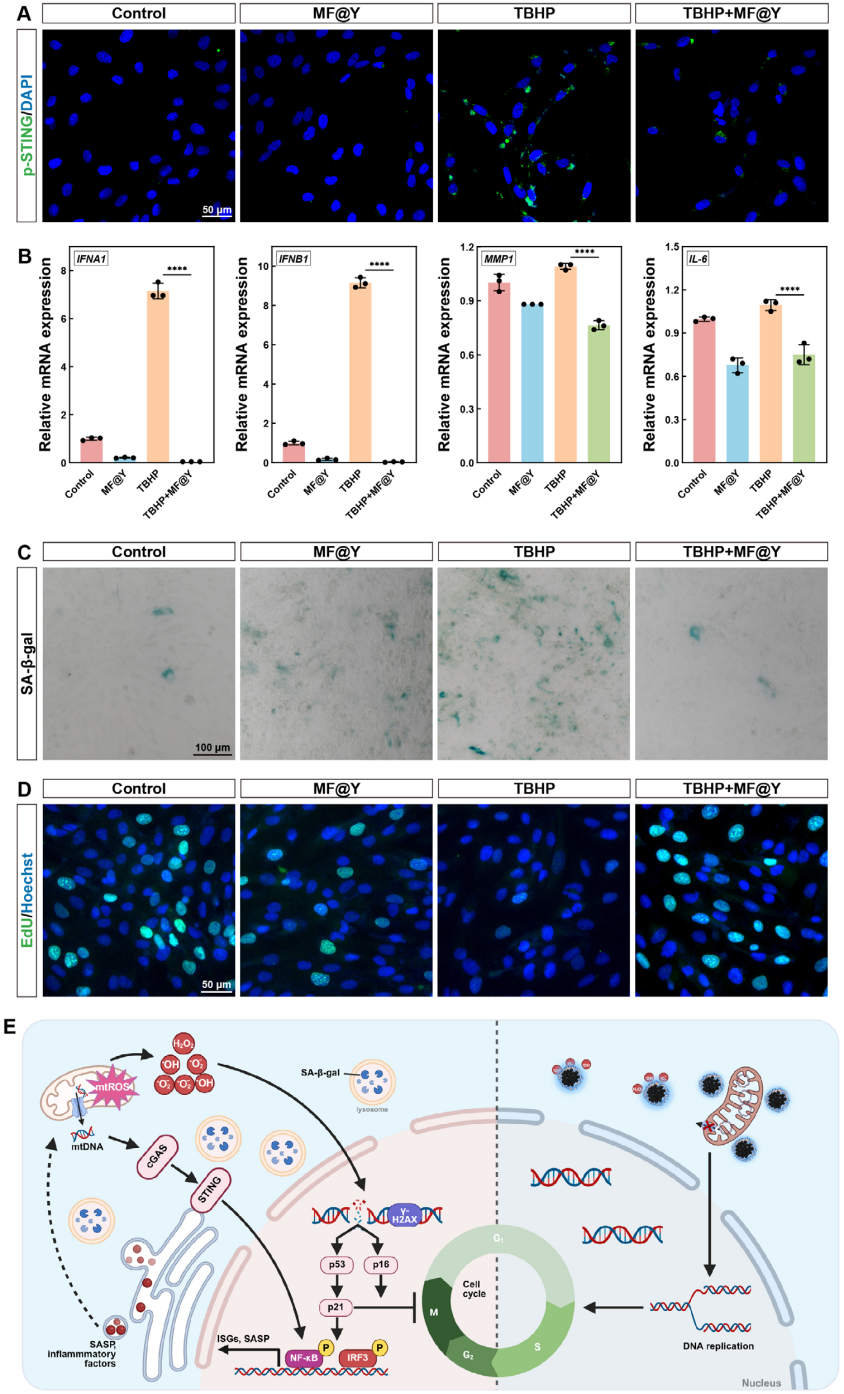
Effects of the MF@Y nanozyme on the cGAS‒STING pathway and antisenescence. (A) Immunofluorescence staining of p‐STING. (B) Relative mRNA expression levels of *IFNA1*, *IFNB1*, and SASP factors (*MMP1* and *IL‐6*). The data are expressed as the mean ± SD (n = 3). ****, *p* < 0.0001 (one‐way ANOVA followed by Tukey's post hoc test). (C) SA‐β‐gal staining after different treatments. TBHP was used to stimulate cellular senescence. (D) EdU staining to evaluate cell proliferation after different treatments. (E) Schematic diagram showing the effects of MF@Y nanozyme on antisenescence. This illustration was created with BioRender.com.

Next, we investigated the ability of the MF@Y nanozyme to rejuvenate senescent cells. First, senescence‐associated β‐galactosidase (SA‐β‐gal) staining was used to assess cellular senescence. As shown in Figure 4C and Figure S5D, there were more β‐galactosidase‐positive cells in the TBHP group than in the control group, indicating that TBHP stimulation accelerated cellular senescence. However, compared with the TBHP group, the TBHP+MF@Y group contained significantly fewer β‐galactosidase‐positive cells. Next, immunofluorescence staining of γ‐H2AX, a recognized biomarker of cellular senescence, was employed to assess the degree of DNA damage. The results revealed extensive green fluorescence in the cell nuclei in the TBHP group, indicative of significant DNA damage (Figure S5E), while fewer green fluorescent nuclei were observed in the TBHP+MF@Y group. Additionally, RT‐qPCR analysis was employed to measure the expression of senescence‐associated genes (*p16* and *p21*). The results confirmed downregulation of the senescence‐associated genes *p16* and *p21* in MF@Y nanozyme‐treated cells relative to the TBHP group (Figure S5F). Finally, 5‐ethynyl‐2‐deoxyuridine (EdU) staining was employed to assess cell proliferation, which can also serve as an indirect indicator of cellular senescence, as senescent cells typically exhibit a diminished proliferative capacity. As depicted in Figure 4D and Figure S5G, the TBHP+MF@Y group presented more green fluorescent nuclei than did the TBHP group. Together, these results indicate that the MF@Y nanozyme significantly mitigates DNA damage, alleviates cellular senescence, and increases cell proliferation (Figure 4E).

### 2.8 Effects of the MF@Y Nanozyme on Endothelial Cells

In the hyperglycaemic microenvironment of diabetes patients, excessive ROS accumulation affects normal cellular functions, such as the migratory capacity of endothelial cells and mesenchymal stem cells and the angiogenic potential of endothelial cells, which significantly impair bone regeneration at diabetic bone defects [55, 56]. Thus, we assessed the impact of the MF@Y nanozyme on cell migration in the presence of high levels of ROS. As shown in Figure 5A and Figure S6A, compared with that in the control group, the migratory capacity of HMEC‐1 human microvascular endothelial cells in the TBHP group was significantly attenuated. Meanwhile, cells in the TBHP+MF, TBHP+Yoda1, and TBHP+MF@Y groups exhibited markedly greater migratory capacity than those in the TBHP group, as more cells crossed the Transwell membrane in the former groups. Notably, the cells in the TBHP+MF@Y group presented the greatest migratory capacity in the presence of high levels of ROS.

**FIGURE 5:**
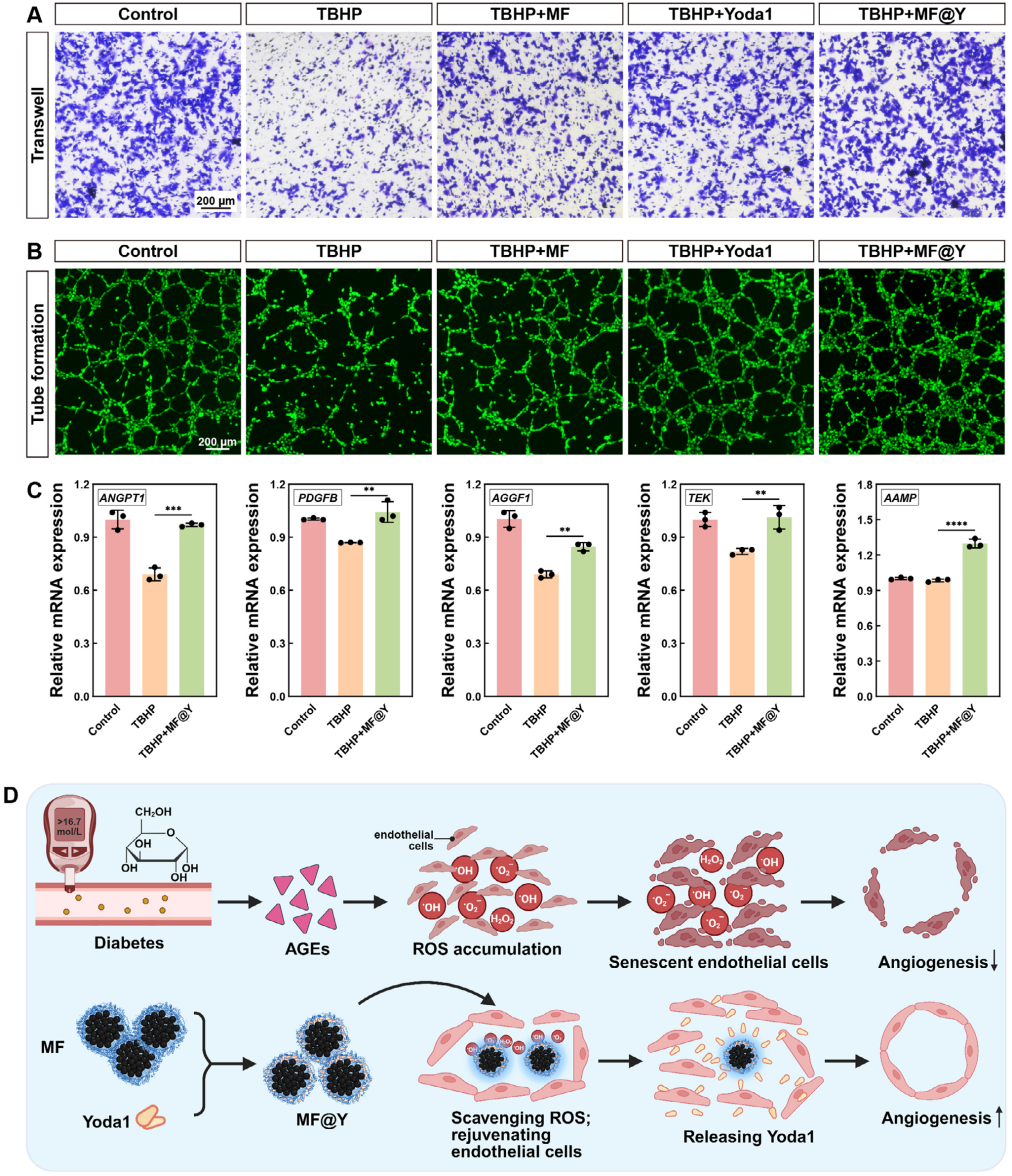
Effects of the MF@Y nanozyme on endothelial cells. (A) Evaluation of the migration of HMEC‐1 cells in the presence of high levels of ROS after different treatments. (B) Tube formation by HMEC‐1 cells in the presence of high levels of ROS after different treatments. (C) Relative mRNA expression levels of angiogenesis‐related genes, including *ANGPT1*, *PDGFB*, *AGGF1*, *TEK*, and *AAMP*. The data are expressed as the mean ± SD (n = 3). **, *p* < 0.01, ***, *p* < 0.001, ****, *p* < 0.0001 (one‐way ANOVA followed by Tukey's post hoc test). (D) Schematic diagram showing the effects of the MF@Y nanozyme on endothelial cells. This illustration was created with BioRender.com.

Furthermore, we evaluated the angiogenic capacity of endothelial cells using tube formation assays. The results shown in Figure 5B and Figure S6B indicate that TBHP led to a notable decrease in the ability of HMEC‐1 cells to migrate and self‐assemble into capillary‐like networks, resulting in the formation of few mesh structures and junctions. However, after treatment with the MF@Y nanozyme, the ability of the cells to form tubes was restored to the most significant extent, and interestingly, we observed that the tube formation ability of cells in the TBHP+Yoda1 group was greater than that of cells in the TBHP+MF group. This likely reflects the role of Yoda1 as a specific agonist of Piezo1, binding directly to its agonist transduction motif. This interaction stabilizes the channel's open conformation, lowers its activation threshold, and antagonizes Piezo1 inactivation induced by elevated ROS levels, thereby restoring endothelial mechanotransduction. This functional recovery enables endothelial cells to sense and respond to mechanical cues in the microenvironment, an essential prerequisite for migration and tube formation, thereby ultimately reversing ROS‐induced impairment of endothelial function.

In addition, we employed RT‐qPCR to assess the effects of the MF@Y nanozyme on the expression of genes related to angiogenesis, including angiopoietin 1 (*ANGPT1*), platelet‐derived growth factor subunit B (*PDGFB*), angiogenic factor with G‐patch and FHA domains 1 (*AGGF1*), TEK receptor tyrosine kinase (*TEK*), and angio‐associated migratory cell protein (*AAMP*), in the presence of high levels of ROS. The results revealed that following TBHP treatment, the expression of angiogenesis‐related genes was inhibited, whereas the MF@Y nanozyme significantly increased expression of these genes (Figure 5C). In conclusion, the MF@Y nanozyme effectively counteracts ROS‐induced impairments in cell migration and angiogenesis (Figure 5D).

### 2.9 The MF@Y Nanozyme Modulates Differentiation of HBMSCs in vitro

BMSCs are non‐hematopoietic bone marrow stem cells with multilineage potential, including differentiation into the osteogenic, chondrogenic, and adipogenic lineages [28]. The induction of osteogenic differentiation and the inhibition of adipocyte differentiation are key in repairing diabetic bone defects [28, 57]. However, BMSC differentiation is affected by various factors, notably high ROS levels in the microenvironment [14, 58]. To investigate the potential of the MF@Y nanozyme to modulate the osteogenic differentiation of HBMSCs, we first evaluated the early osteogenic differentiation of HBMSCs in the presence of high levels of ROS using alkaline phosphatase (ALP) staining. As shown in Figure 6A and Figure S7A, the high ROS environment created by TBHP treatment significantly suppressed ALP expression in HBMSCs. However, ALP was significantly upregulated in the MF, Yoda1, and MF@Y nanozyme groups compared with the TBHP group. Notably, the TBHP+MF@Y group exhibited the most pronounced increase in ALP expression, followed by the TBHP+Yoda1 and TBHP+MF groups.

**FIGURE 6:**
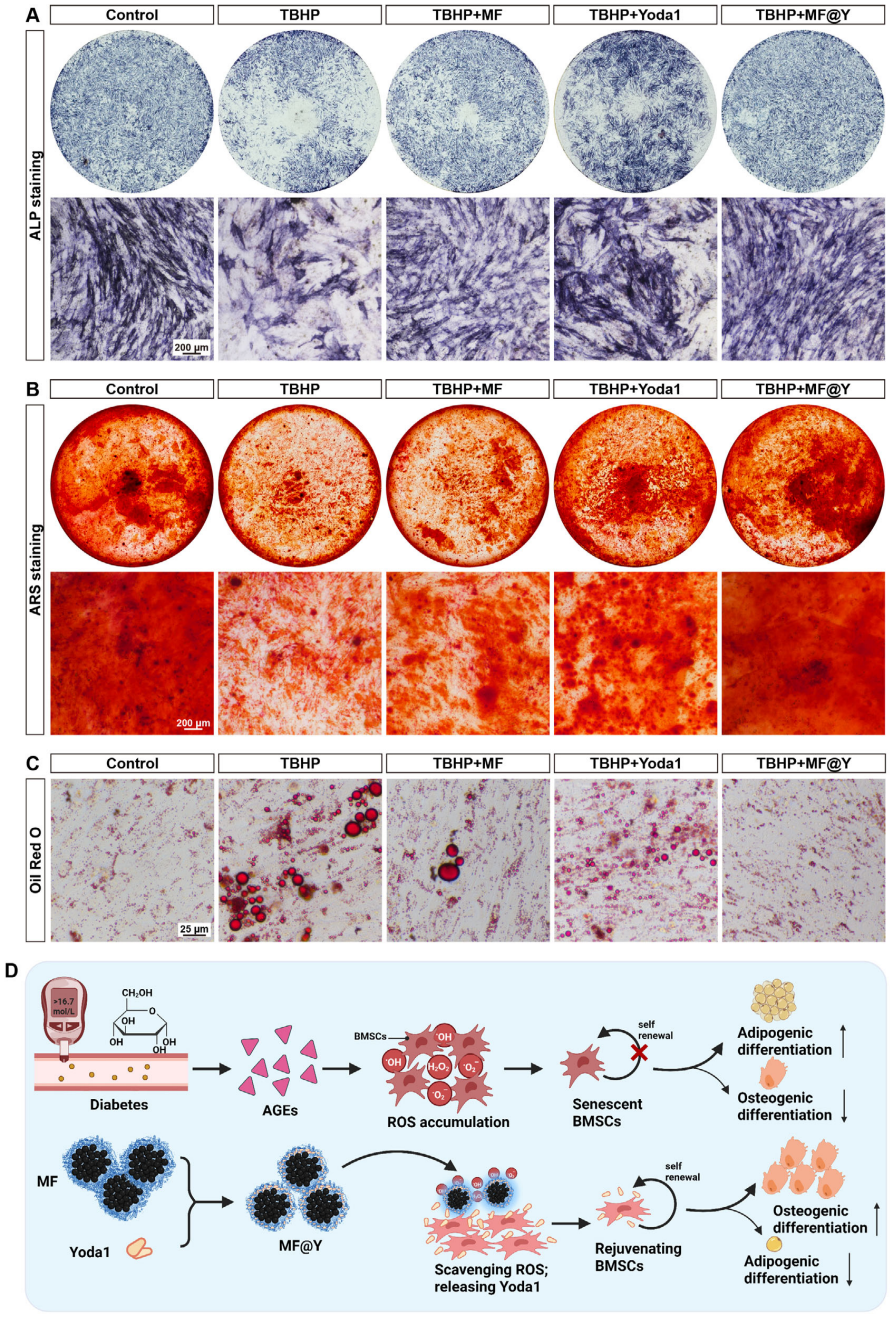
Effects of the MF@Y nanozyme on differentiation of HBMSCs. (A) Representative gross and microscopic images of ALP staining to assess the osteogenic ability of the HBMSCs. (B) Representative gross and microscopic images of ARS staining to assess the osteogenic differentiation of the HBMSCs. (C) Oil Red O staining images to assess the adipogenic differentiation of the HBMSCs. (D) Schematic diagram showing the effects of MF@Y nanozyme on HBMSCs differentiation. This illustration was created with BioRender.com.

Next, alizarin red S (ARS) staining was employed to assess calcium nodule formation, a hallmark of the terminal stage of osteogenic differentiation. Consistent with the ALP staining results, TBHP treatment resulted in only sparse areas of calcium nodules, whereas the MF nanozyme, Yoda1, and MF@Y nanozyme groups displayed increased calcium nodule formation (Figure 6B and Figure S7B). Notably, among all the high ROS groups, the MF@Y nanozyme‐treated group presented the largest and most intensely stained calcium nodules. These findings indicate that TBHP significantly inhibited the osteogenic differentiation of HBMSCs, whereas osteogenic differentiation was partially restored upon MF nanozyme and Yoda1 treatment. Importantly, the MF@Y nanozyme induced the most pronounced increase in osteogenic differentiation.

RT‐qPCR was then performed to evaluate the impact of the MF@Y nanozyme on the expression of osteogenesis‐related genes, including osteocalcin (*OCN*), *ALP*, and type I collagen A1 (*COL1A1*). As shown in Figure S7C, under normal conditions, the expression of osteogenesis‐related genes was greater in the MF@Y nanozyme group than in the control group. Furthermore, in the presence of high levels of ROS, the expression levels of osteogenesis‐related genes in the TBHP+MF@Y group were higher than those in the TBHP group. These results indicated that the MF@Y nanozyme significantly increases the expression of osteogenesis‐related genes under both normal and high‐ROS conditions.

In addition, we used Oil Red O staining to assess adipogenic differentiation. As shown in Figure 6C and Figure S7D, compared with normal conditions (control), high‐ROS conditions (in the TBHP group) significantly promoted the differentiation of HBMSCs into adipocytes and the formation of numerous lipid droplets. However, upon treatment with the MF nanozyme, Yoda1, or the MF@Y nanozyme, the size and number of lipid droplets significantly decreased, with the TBHP+MF@Y group presenting the fewest and smallest lipid droplets, followed by the TBHP+Yoda1 group and the TBHP+MF group. To further characterize these effects at the molecular level, RT‐qPCR was performed to assess the effects of MF@Y nanozymes on the expression of adipogenesis‐associated genes, including Lipoprotein lipase (*LPL*), CCAAT/enhancer‐binding protein alpha (*C/EBPα*), *C/EBPβ*, and Adiponectin (*ADIPOQ*). As shown in Figure S7E, under high ROS conditions, MF@Y nanozyme treatment significantly attenuated the expression of these pro‐adipogenic genes, suggesting that the antioxidant activity of the nanozyme is sufficient to override ROS‐driven adipogenic signaling and suppress aberrant lipogenic commitment. Collectively, these results demonstrate that elevated ROS promotes adipogenic differentiation of HBMSCs while simultaneously suppressing osteogenic commitment; critically, MF@Y nanozyme treatment reverses this imbalance by restoring osteoblast differentiation and inhibiting adipogenesis (Figure 6D).

### 2.10 Inhibitory Effects of the MF@Y Nanozyme on Osteoclast Differentiation In Vitro

Diabetes and osteoporosis often coexist due to excessive ROS production, which disrupts BMSCs function and inhibits bone formation while also increasing osteoclast activity and bone resorption [26, 59]. Therefore, suppressing osteoclast activation is key to promoting the healing of diabetic bone defects. First, we employed staining of tartrate‐resistant acid phosphatase (TRAP), a specific marker of osteoclasts, to assess the inhibition of osteoclast differentiation in the presence of high levels of ROS, which can be assessed by observing purple osteoclasts. As shown in Figure 7A and Figure S8A, after incubation in osteoclastogenic induction medium for 7 days, the TBHP group contained more TRAP‐positive cells than did the control group. In addition, among the high ROS groups, the TBHP+MF@Y group presented the lowest number of TRAP‐positive cells, followed by the TBHP+MF, TBHP+Yoda1, and TBHP groups. F‐actin and 4’,6‐diamidino‐2‐phenylindole (DAPI) staining were subsequently performed to label osteoclasts and assess multinucleation and actin ring development. As shown in Figure 7B and Figure S8B, in the presence of high levels of ROS, the TBHP group presented the greatest number of very large, multinucleated cells, followed by the TBHP+Yoda1 group and the TBHP+MF group, confirming the TRAP staining results. Notably, in the presence of the MF@Y nanozyme, few very large multinucleated cells with fluorescent actin rings were observed. These results indicate that high ROS levels can significantly promote osteoclast differentiation, whereas the MF@Y nanozyme markedly inhibits this effect and suppresses osteoclast differentiation.

**FIGURE 7:**
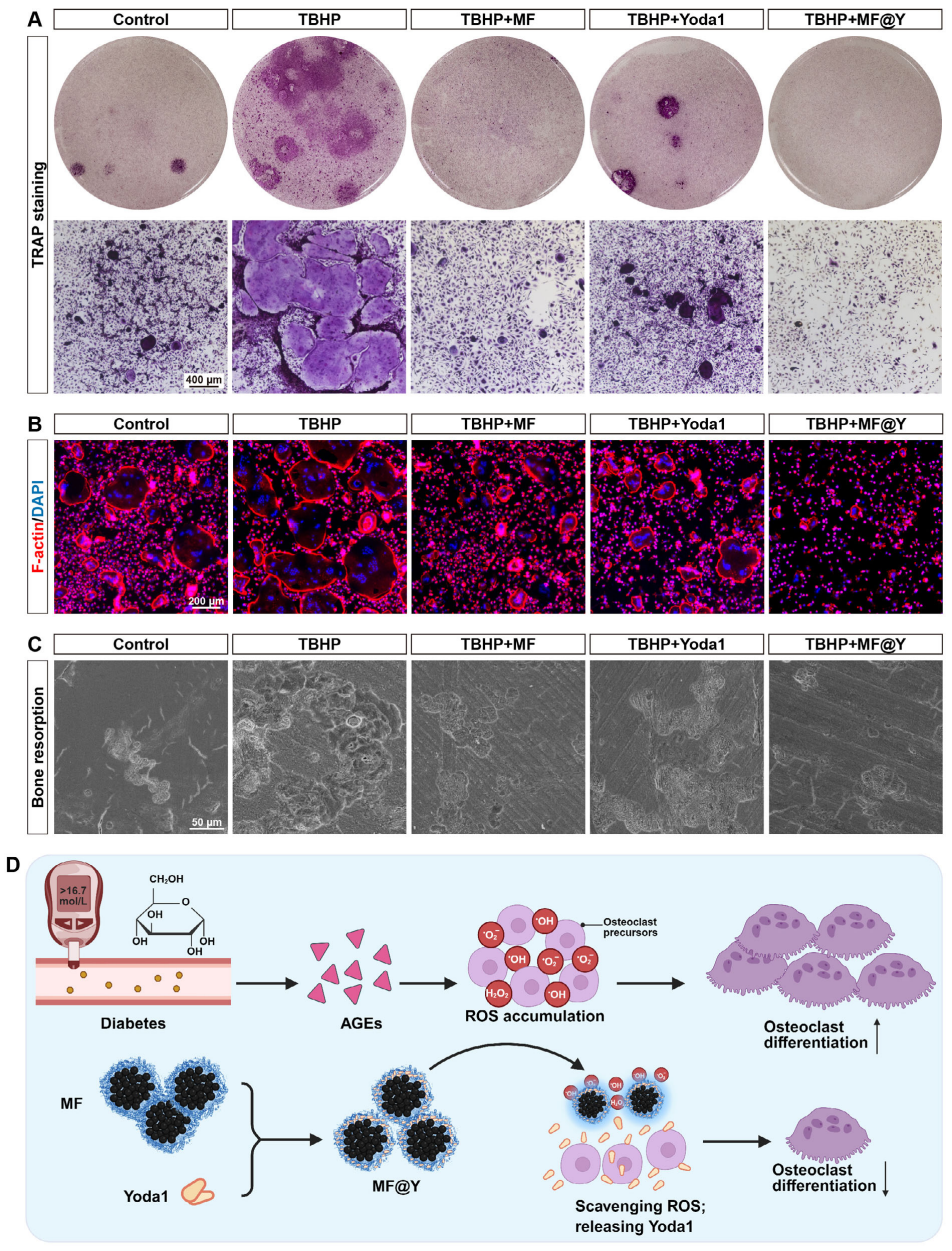
Inhibitory effects of the MF@Y nanozyme on osteoclast differentiation in vitro. (A) Representative gross and microscopic images of TRAP staining to evaluate osteoclast differentiation. (B) Fluorescence images of osteoclasts. (C) SEM images of resorption lacunae formed by osteoclasts. (D) Schematic diagram showing effects of the MF@Y nanozyme on osteoclast differentiation. This illustration was created with BioRender.com.

Next, the bone resorption ability of osteoclasts was assessed by culturing osteoclasts on bone slices (Figure 7C and Figure S8C). The bone slices in the TBHP group presented many deep resorption lacunae, whereas the areas and depths of the resorption lacunae were lower in the other high ROS groups than in the TBHP group, with the TBHP+MF@Y group exhibiting the most pronounced decrease. These data indicate that in the presence of high levels of ROS, the MF@Y nanozyme suppresses the differentiation and bone resorption abilities of osteoclasts (Figure 7D).

### 2.11 The MF@Y Nanozyme Regulates Macrophage Polarization In Vitro

Macrophage polarization plays a pivotal role in tissue regeneration, with M1 macrophages driving inflammation to clear debris and initiate repair, whereas M2 macrophages promote tissue repair and remodeling through the secretion of growth factors [60, 61, 62, 63]. In the context of normal bone defect healing, the balance between M1 and M2 macrophages is crucial for effective bone regeneration. However, in diabetes patients, the hyperglycaemic microenvironment disrupts macrophage polarization, resulting in a predominance of M1 macrophages that secrete proinflammatory cytokines, thereby perpetuating chronic inflammation and hindering bone repair [64, 65]. Hence, modulation of macrophage polarization, specifically inhibition of M1 macrophage polarization and promotion of M2 macrophage polarization, is critically important in diabetic bone defect repair. First, immunofluorescence staining of iNOS (a marker of M1 macrophages) and CD206 (a marker of M2 macrophages) was used to assess macrophage polarization. As shown in Figure 8A and Figure S9, more red fluorescent cells were observed in the TBHP and TBHP+Yoda1 groups than in the control group, whereas few red fluorescent cells were observed in the TBHP+MF and TBHP+MF@Y groups. Moreover, as shown in Figure 8A and Figure S9, few CD206‐positive cells were observed in the TBHP group, whereas many CD206‐positive cells were observed in the TBHP+MF and TBHP+MF@Y groups. Interestingly, compared with the TBHP group, the TBHP+Yoda1 group presented more CD206‐positive cells.

**FIGURE 8:**
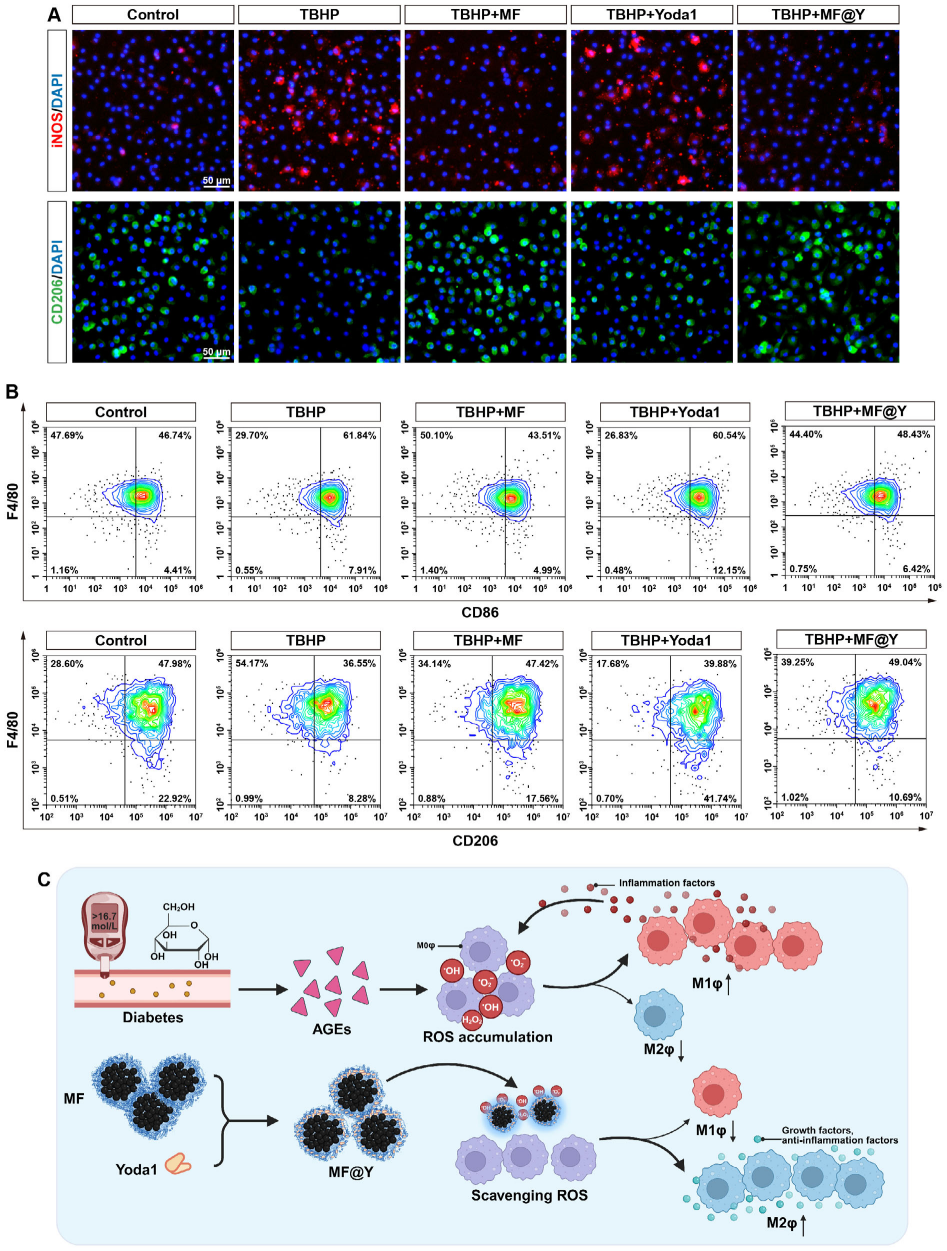
Ability of the MF@Y nanozyme to regulate macrophage polarization in vitro. (A) Immunofluorescence staining of iNOS (M1 macrophages) and CD206 (M2 macrophages) to evaluate macrophage polarization. (B) Flow cytometric analysis of macrophage polarization. M1 macrophages: F4/80^+^ and CD86^+^; M2 macrophages: F4/80^+^ and CD206^+^. (C) Schematic diagram showing effects of the MF@Y nanozyme on macrophage polarization. This illustration was created with BioRender.com.

Next, flow cytometry was performed to assess macrophage polarization, with CD86 used to label M1 macrophages and CD206 used to label M2 macrophages. As shown in Figure 8B, the TBHP group presented the highest percentage of M1 macrophages (61.84%) and the TBHP+MF group presented the lowest percentage (43.51%), followed by the control group (46.74%), the TBHP+MF@Y group (48.43%), and the TBHP+Yoda1 group (60.54%). Furthermore, the TBHP+MF@Y group presented the highest percentage of M2 macrophages (49.04%), followed by the control group (47.98%), the TBHP+MF group (47.42%), the TBHP+Yoda1 group (39.88%), and the TBHP group (36.55%). These data indicate that high ROS levels significantly promote M1 macrophage polarization and inhibit M2 macrophage polarization; additionally, Yoda1 seemed to have no effect on the inhibition of M1 macrophage polarization but exhibited a minor ability to promote M2 macrophage polarization in the presence of high levels of ROS. Moreover, the MF@Y nanozyme significantly reversed the effects of ROS (Figure 8C).

### 2.12 Characterization of the BCPG‐MF@Y Scaffold

After thoroughly evaluating the effects and mechanisms of the MF@Y nanozyme, we proceeded to construct the BCPG‐MF@Y scaffold for functional integration. The fabrication process began with three‐dimensional printing of the BCP scaffold using DLP technology. First, micro‐CT imaging confirmed the structural integrity of the scaffold, revealing a precisely engineered diamond cubic lattice configuration (Figure S10A). Subsequent scanning electron microscopy (SEM) analysis provided critical insights into the microarchitecture of the scaffold. Both surface and cross‐sectional views consistently revealed an intricate network of interconnected porous structures within the fibrous matrix (Figure S10B).

Building upon the structural characterization of the BCP scaffold, we focused on optimizing drug delivery by integrating pGelMA hydrogels. pGelMA was chosen over conventional GelMA hydrogels owing to its distinct advantages of enhanced biomimetic porosity, which better mimics the extracellular matrix [66, 67, 68], and superior angiogenic and osteogenic potential due to its expanded pore architecture, which facilitates cellular infiltration and metabolic exchange [69]. We then evaluated the structure and morphology of the pGelMA hydrogels via confocal microscopy after labeling the pGelMA hydrogels with a fluorescent dye. As shown in Figure S10C, the hydrogels contained pores with a diameter of approximately 100 µm. In addition, SEM was employed to observe hydrogel cross‐sections. As shown in Figure S10D, the hydrogels exhibited a highly porous structure. These results indicated that the pGelMA hydrogels feature a highly porous structure, which may facilitate cell migration and nutrient transport.

Given the observed structural advantages of pGelMA hydrogels in supporting cellular activities, we further investigated their biocompatibility with the engineered nanozyme systems. Specifically, biocompatibility assessments of the pGelMA, MF nanozyme‐incorporated pGelMA‐MF, and MF@Y nanozyme‐loaded pGelMA‐MF@Y hydrogels were conducted. To assess cellular responses, HBMSCs were cultured on these hydrogels for 5 or 7 days, after which Live/Dead cell staining was performed to evaluate cell viability and proliferation. As shown in Figure S10E, all the hydrogels exhibited excellent biocompatibility, and few cell nuclei were stained with propidium iodide (PI; red fluorescence, indicating dead cells). In addition, over time, the HBMSCs gradually expanded within the hydrogels. These data suggest that the pGelMA, pGelMA‐MF, and pGelMA‐MF@Y hydrogels exhibited excellent biocompatibility and promoted cell growth.

Next, we combined the BCP scaffold with the pGelMA hydrogels to construct a BCPG scaffold and integrated the BCP scaffold with the pGelMA‐MF and pGelMA‐MF@Y hydrogels separately to create the BCPG‐MF and BCPG‐MF@Y scaffolds, respectively. To characterize the release kinetics of the BCPG‐MF@Y scaffold under physiologically relevant conditions, we systematically monitored mass loss (Figure S10F), drug release profiles (Figure S10G), zeta potential (Figure S10H), and supernatant pH (Table S1) over the course of in vitro degradation. Both the MF nanozyme and Yoda1 exhibited sustained, gradual release throughout this process, persisting over approximately six weeks. Notably, the zeta potential of the supernatant remained stable at approximately +9 mV, and the pH was maintained at approximately 7.0 throughout the observation window. Then, we assessed cell adhesion and growth on these scaffolds. As shown in Figure S10I, a substantial number of cells adhered to and proliferated on the scaffolds. Compared with those on the BCP scaffold, the cells on the BCPG, BCPG‐MF, and BCPG‐MF@Y scaffolds exhibited more spreading and outgrowth, indicating that the pGelMA hydrogels facilitated cell growth and proliferation.

### 2.13 Effects of BCPG‐MF@Y In Vivo

Following the systematic in vitro evaluations, subsequent in vivo assessments of the osteogenic efficacy of the BCPG‐MF@Y scaffold were conducted in a diabetic rat model with a 5 mm critical‐size calvarial defect that cannot heal via intrinsic bone regeneration. To characterize the in vivo degradation of the scaffold components, we labeled the pGelMA hydrogel with Cy7 fluorescent dye. As shown in Figure S11A, the hydrogel coating of the BCPG‐MF@Y scaffold was almost completely degraded within approximately 5 weeks. Twelve weeks after the scaffolds were implanted in the cranial defects, the rat skulls were collected, and micro‐CT analysis was employed to evaluate new bone formation. As shown in Figure 9A and Figure S11B, the 3D reconstructions and cross‐sectional images revealed minimal bone regeneration in the no‐scaffold (control) group. Additionally, the BCP and BCPG scaffold groups presented limited new bone formation, whereas the BCPG‐MF and BCPG‐MF@Y groups exhibited substantial new bone formation. Notably, bone regeneration was greatest in the BCPG‐MF@Y group. These data indicate that critical‐size cranial defects do not heal in the absence of a scaffold, the BCP and BCPG scaffolds facilitate bone defect healing to a limited degree, and the BCPG‐MF@Y scaffold demonstrates a significantly greater capacity to promote bone regeneration in a diabetic rat bone defect model.

**FIGURE 9:**
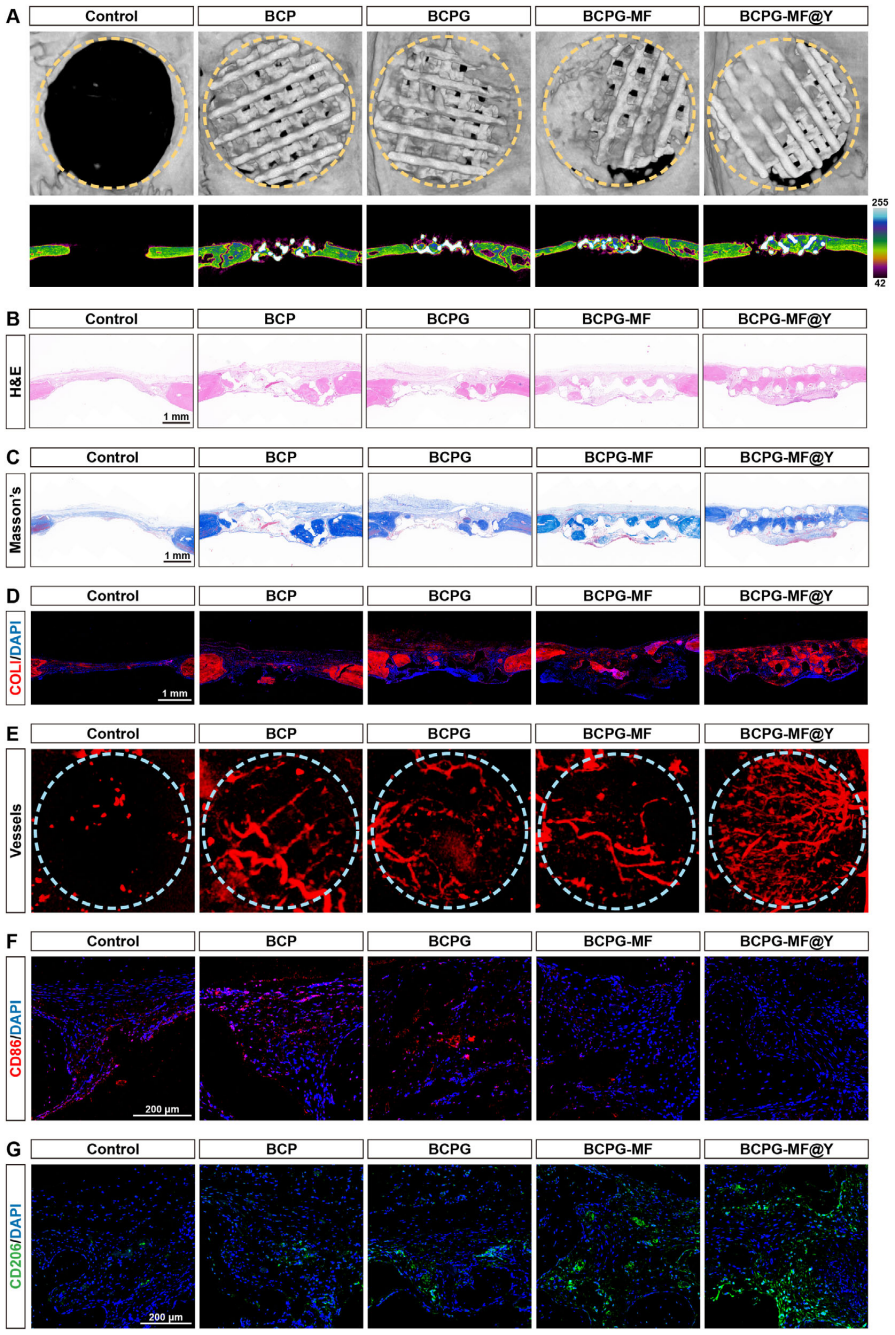
Ability of the BCPG‐MF@Y scaffold to promote bone regeneration and angiogenesis and regulate macrophage polarization in vivo. (A) Micro‐CT images of the scaffolds 12 weeks after implantation. (B) H&E‐stained images of calvarial bone defect tissue sections. (C) Masson's trichrome‐stained images of calvarial bone defect tissue sections. (D) Immunofluorescence images of COLI. (E) Micro‐CT images of newly formed vessels 7 weeks after scaffold implantation. (F) Images of immunofluorescence staining of CD86 to detect M1 macrophages. (G) Images of immunofluorescence staining of CD206 to detect M2 macrophages.

Hematoxylin and eosin (H&E) staining (Figure 9B and Figure S11C) and Masson's trichrome staining (Figure 9C and Figure S11D) were subsequently performed to evaluate new bone formation and collagen deposition. The results revealed the greatest extents of bone regeneration and collagen deposition in the BCPG‐MF@Y group, followed by the BCPG‐MF, BCPG, and BCP groups. Notably, substantial new bone formation was evident across the entirety of the BCPG‐MF@Y scaffold, whereas new bone formation was primarily observed at the periphery of the other scaffolds, with minimal bone formation observed in the central region. Furthermore, immunofluorescence staining of COLI was employed to evaluate bone regeneration. Consistent with the results of H&E and Masson's trichrome staining, the BCPG‐MF@Y group presented the greatest extent of collagen formation and maturation, followed by the BCPG‐MF, BCPG, and BCP groups (Figure 9D). These findings indicate that BCPG‐MF@Y significantly enhances osteogenic activity and collagen matrix formation in a diabetic rat bone defect model.

Given the pivotal role of angiogenesis in bone regeneration, angiogenesis was subsequently evaluated following implantation of the BCPG‐MF@Y scaffold into diabetic rat bone defects. At 7 weeks post‐implantation, Microfil perfusion was employed to label blood vessels, and micro‐CT was utilized to visualize neovascularization. As shown in Figure 9E, minimal neovascularization was observed in the untreated control group, whereas scaffold implantation markedly promoted new vessel ingrowth across all groups. Notably, the BCPG‐MF@Y group exhibited the most extensive neovascularization, with a significantly greater vessel volume and connectivity relative to all other groups. To further corroborate these findings, immunofluorescence staining for the endothelial marker CD31 was performed on sections harvested from the defect site. Consistent with the micro‐CT results, CD31 staining revealed markedly enhanced vascular density in the BCPG‐MF@Y group compared with all other groups (Figure S11E), collectively demonstrating that the synergistic combination of scaffold components in BCPG‐MF@Y potently drives angiogenesis within the diabetic bone defect microenvironment.

We further assessed macrophage polarization in vivo using immunofluorescence staining of CD86 and CD206 to label M1 and M2 macrophages, respectively. As shown in Figure 9F and S11F, the control, BCP, and BCPG groups included many CD86‐positive cells in the bone defects, while there were few CD86‐positive cells in the BCPG‐MF and BCPG‐MF@Y groups, with the BCPG‐MF@Y group presenting the fewest CD86‐positive cells. Moreover, as shown by immunofluorescence staining, the BCPG‐MF@Y group presented the greatest number of CD206‐positive cells, followed by the BCPG‐MF, BCPG, BCP, and control groups (Figure 9G and Figure S11G). These data indicate that the BCPG‐MF@Y scaffold can regulate macrophage polarization in vivo by inhibiting M1 macrophage polarization and promoting M2 macrophage polarization.

In addition, to evaluate systemic biosafety, major organs (heart, liver, spleen, lung, and kidney) were harvested at 12 weeks post‐implantation and processed for H&E staining. Histological examination (Figure S12) revealed no evidence of inflammatory infiltration, necrosis, fibrosis, or structural abnormalities in any scaffold‐implanted group relative to controls, indicating that none of the implants elicited systemic toxicity or adverse immune responses over the course of the study.

## 3 Discussion

This study targets the core obstacles to diabetic bone repair, namely excessive mtROS production, cellular senescence, and chronic inflammation, by constructing a BCPG‐MF@Y multifunctional scaffold. This system integrates mitochondrial protection, Piezo1‐mediated mechano‐signaling activation, and osteoimmune remodeling. By leveraging Yoda1‐functionalized biomimetic nanozymes to synergistically modulate multiple pathways, the scaffold efficiently reprograms the pathological microenvironment of diabetic bone defects and achieves vascularized bone regeneration, demonstrating marked innovative advantages and clinical translation potential over existing therapeutic systems.

Current systems for treating diabetic bone defects often target single pathological pathways, failing to interrupt the hyperglycemia‐driven cascade of tissue damage. Standalone biological scaffolds provide structural support and osteoconduction but cannot reverse mitochondrial dysfunction, cellular senescence, or immune dysregulation induced by hyperglycemia [70]; nanozyme‐based systems alone are unable to specifically block the mtDNA–cGAS–STING inflammatory signaling axis, leaving the upstream sources of inflammation and senescence unaddressed [71]; reported Yoda1‐based systems focus solely on osteogenic effects without achieving multidimensional functional integration, precluding multi‐target synergistic intervention in complex pathological processes [34, 72]. Existing periodontal regeneration strategies for periodontitis face analogous limitations. Bone graft materials, whether autograft, allograft, xenograft, or synthetic BCP, and single growth factor delivery vehicles such as BMP‐2‐loaded collagen membranes, primarily offer physical support or isolated osteogenic stimuli, while neglecting critical barriers including immune‐inflammatory imbalance, ROS accumulation, and insufficient vascularisation, resulting in limited efficacy and high recurrence rates [73, 74]. While advanced systems incorporate anti‐inflammatory agents (e.g., IL‐4‐ or IL‐10‐loaded microspheres) or antioxidant components (e.g., CeO_2_ nanozymes), or utilize hydrogels to modulate the local microenvironment, these approaches typically enhance single functions or lack effective integration with structural scaffolds, hindering spatiotemporal coordination of immunomodulation, osteogenesis, and angiogenesis [75, 76]. Therapeutic outcomes are even less favorable in the complex microenvironment of diabetes complicated by periodontitis [77, 78]. To address these conceptual and design limitations, in the present study we developed a BCPG‐MF@Y scaffold, constructing a dual‐porosity structure that combines macroscopic mechanical support with a microscopic biomimetic microenvironment, providing both stable spatial maintenance for defect sites and an adapted mechanical niche for Piezo1 channel activation, thereby achieving synergistic coupling of structural mechanics and biochemical signals. The scaffold delivers dual core therapeutic actions. Yoda1‐mediated Piezo1 activation enhances osteogenic and angiogenic differentiation while suppressing adipogenesis and osteoclastogenesis. Meanwhile, MF nanozymes restore mitochondrial homeostasis, block the mtDNA–cGAS–STING axis, and promote macrophage polarization toward a reparative phenotype to remodel osteoimmune homeostasis, thereby mitigating senescence and scavenging excess mtROS (Figure 10). Synergistically, these effects and the enhanced matrix maturation demonstrate the microenvironmental reprogramming needed for diabetic bone regeneration.

**FIGURE 10:**
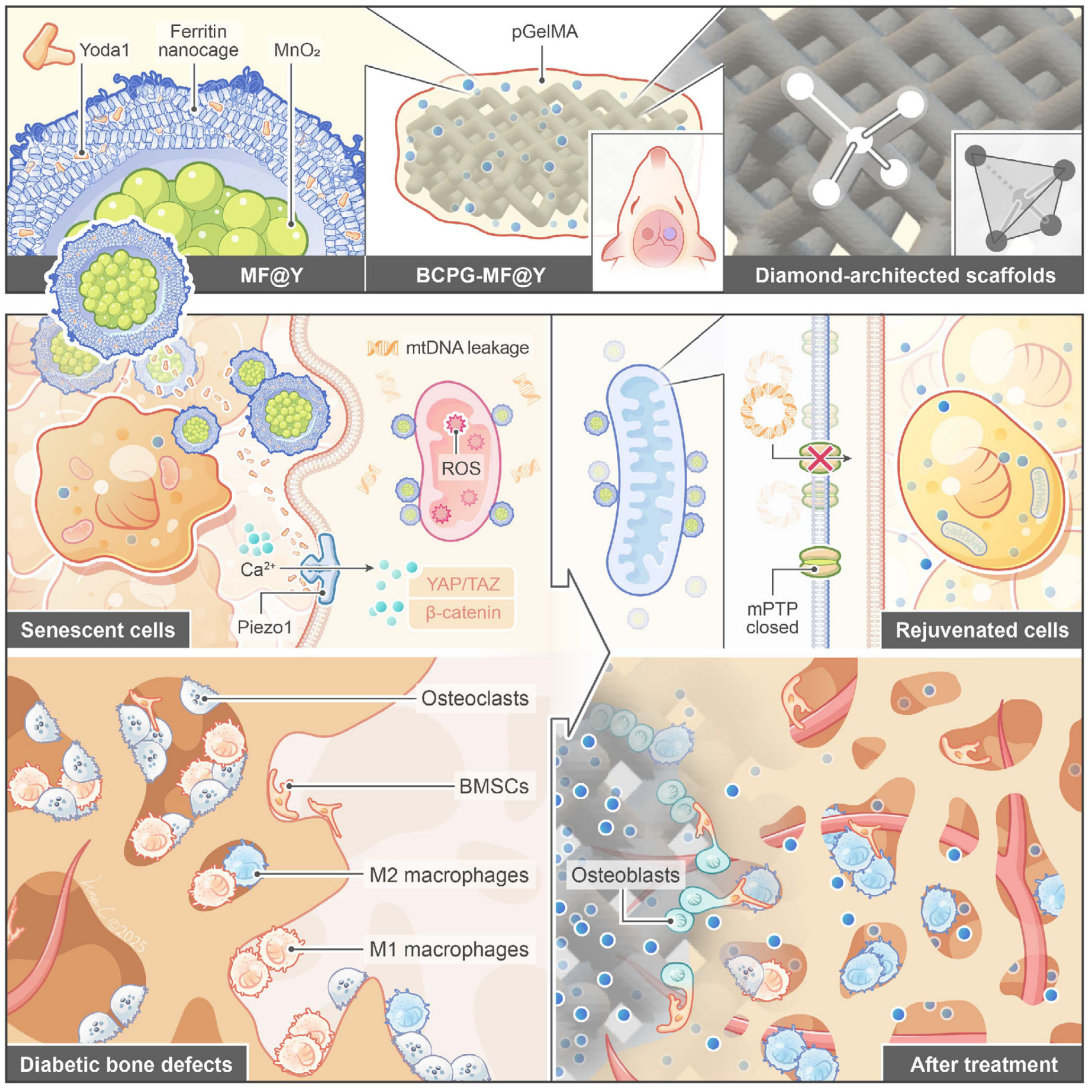
Schematic diagram showing the mechanism by which the BCPG‐MF@Y scaffold promotes bone regeneration in diabetic rats. Illustration credit: Lina Cao.

Within the BCPG‐MF@Y scaffold system, the BCP component affords long‐term mechanical support and an osteoconductive/osteoinductive microenvironment for bone defects, while the pGelMA hydrogel functions as a controlled‐release vehicle for MF@Y. During fabrication, pre‐polymerized pGelMA solution infiltrates BCP pores, and subsequent photocrosslinking anchors the resulting pGelMA network within the BCP scaffold via physical interlocking. This anchoring is critical for preventing carrier detachment during initial implantation. Additional potential physicochemical interactions, such as ionic bonding between BCP surface ions (Ca^2+^, PO_4_^3−^) and pGelMA functional groups (─NH_2_, ─COOH), may further reinforce interfacial integration. The degradation kinetics of pGelMA and BCP differ markedly and are deliberately exploited in this design. Enzymatic and hydrolytic degradation of pGelMA underpins its role as a drug carrier. Its rapid degradation drives an early burst release of MF@Y, ensuring timely delivery of the MF nanozyme to maintain mitochondrial homeostasis while simultaneously releasing Yoda1 to activate Piezo1‐mediated mechanosensing, both of which are essential for meeting the metabolic and signaling demands of early diabetic bone regeneration. Concurrently, swift pGelMA degradation creates space for host cell migration (osteoblasts, vascular endothelial cells), proliferation, and tissue ingrowth, thereby preventing physical obstruction and enhancing BCP osteoinductive functionality. By contrast, the BCP scaffold independently maintains long‐term structural support owing to its intrinsic mechanical strength and slow degradation profile. Even after early pGelMA degradation, BCP preserves structural stability and mechanical integrity at the defect site, resisting load and preventing collapse. Its sustained degradation ensures scaffold integrity throughout regeneration, providing a stable template for new bone deposition, maturation, and remodeling. Critically, in vivo results (histology and micro‐CT) demonstrate that despite rapid pGelMA degradation, the BCPG‐MF@Y composite scaffold significantly outperforms BCP alone in bone repair outcomes (new bone volume, osseointegration). This finding indicates that material “heterogeneity” in degradation kinetics represents deliberate functional temporal coordination within this design: pGelMA strategically facilitates initial drug delivery and stimulation before degrading, subsequently yielding space for BCP and nascent tissue to orchestrate long‐term structural support and reconstruction.

Mechanotransduction plays a pivotal role in bone regeneration by orchestrating cellular behaviors and signaling pathways through multiple mechanisms, thereby serving as a critical driver of bone defect repair [33, 79]. Among the mechanosensitive elements, the Piezo1 ion channel has emerged as a central mechanotransducer in osteocytes that converts mechanical stress into calcium influx to initiate electrochemical signaling cascades [36]. The Piezo1 channel coordinates with bone homeostasis through the synergistic regulation of BMSCs, osteoblasts, osteocytes, and the vascular endothelium [33]. Precise modulation of Piezo1 activity is particularly important for diabetic bone regeneration, where the impaired healing capacity necessitates targeted therapeutic strategies. Current diabetic bone regeneration approaches primarily employ pharmacological activation or mechanical stimulation, with pharmacological intervention demonstrating superior clinical potential [80, 81]. Unlike mechanical methods requiring sustained external input, molecular targeting enables direct mechanobiological coupling, offering enhanced repair efficiency, broader applicability, and improved spatiotemporal control, attributes essential for reconstructing complex defects in pathologically compromised microenvironments [82]. Notably, Yoda1 has been validated as a potent Piezo1 agonist capable of increasing mechanotransduction [83]. However, its clinical translation faces two challenges: its inherent hydrophobicity, which leads to its rapid degradation and suboptimal pharmacokinetics, coupled with conventional intravenous administration that causes transient supraphysiological concentrations. Such uncontrolled delivery risks Piezo1 overactivation, potentially exacerbating cellular senescence [72]. Previous attempts to integrate Yoda1 into scaffold matrices through direct blending have proven inadequate [72], as material viscosity and mixing heterogeneity induce uneven drug distribution, whereas degradation‐dependent release kinetics result in initial burst release followed by therapeutic insufficiency. To address these limitations, we engineered a hierarchical delivery system leveraging the hydrophobic pockets of the MF nanozyme for Yoda1 encapsulation (MF@Y), which was subsequently incorporated into pGelMA hydrogels. This innovative design achieves enhanced drug dispersion through molecular confinement, minimized concentration gradients, sustained release from hydrogels and the MF@Y nanozyme, and synergistic therapeutic effects. Furthermore, compared with conventional formulation optimization (integrating drugs into a scaffold) requiring extensive trial and error, our modular platform significantly reduces development costs while ensuring manufacturing reproducibility. This study establishes a paradigm‐shifting strategy for hydrophobic drug delivery in bone regeneration, demonstrating particular promise for managing diabetic bone defects where precise microenvironmental control is paramount. The operational simplicity and material efficiency of this methodology underscore its translational potential for clinical adaptation.

Diabetes mellitus, characterized by chronic hyperglycemia, profoundly impairs fracture healing. Substantial evidence indicates that the hyperglycaemic microenvironment disrupts bone repair at multiple metabolic levels, principally by suppressing energy metabolism pathways essential to the regenerative process, including glycolytic flux, mitochondrial function, and ATP synthesis [84, 85]. These bioenergetic deficits propagate downstream biological dysfunction, ultimately delaying bone regeneration. In osteoblasts residing at fracture sites in diabetic subjects, the expression of key glycolytic enzymes, namely hexokinase 2, phosphofructokinase, and pyruvate kinase M2, is markedly downregulated, resulting in attenuated glycolytic activity and insufficient ATP generation [86, 87]. As glycolysis represents a principal route of rapid cellular energy acquisition, its impairment directly curtails osteoblast proliferation, differentiation and mineralization. These processes are inherently energy‐intensive, requiring substantial ATP to sustain collagen synthesis, hydroxyapatite deposition, and extracellular matrix remodeling [88]. In this study, consistent with this, micro‐CT quantification alongside histological analysis revealed that the BCPG‐MF@Y group exhibited marked improvements in both bone quality and maturity relative to other groups, findings that functionally reflect this material's capacity to restore cellular energy metabolism. Energy metabolism defects also critically impair angiogenesis, a highly energy‐demanding process requiring ample ATP for endothelial cell proliferation, migration, and tubulogenesis [89]. Under hyperglycaemic conditions, both glycolysis and oxidative phosphorylation in endothelial cells are suppressed, leading to reduced vascular endothelial growth factor expression, diminished endothelial nitric oxide synthase (eNOS) activity, and increased vascular permeability [90, 91]. To interrogate this dimension experimentally, we employed perfusion‐based micro‐CT to assess vascular density within the bone defect region. Vascular density in the BCPG‐MF@Y group was significantly greater than that observed in the diabetic control and all other groups, strongly implicating the ability of this material to attenuate energy metabolic dysfunction within the hyperglycaemic milieu. Taken together, these observations are consistent with published literature documenting hyperglycemia‐induced bioenergetic dysregulation, and collectively support the notion that disrupted energy metabolism constitutes a central pathological mechanism underlying impaired bone healing in diabetes. Our findings suggest that BCPG‐MF@Y modulates energy‐sensing pathways, plausibly through amelioration of the local microenvironment and attenuation of oxidative stress, thereby restoring cellular energy homeostasis and facilitating bone regeneration under diabetic conditions.

Mitochondria serve as central regulators of cellular homeostasis by orchestrating fundamental biological processes, including oxidative phosphorylation (OXPHOS), metabolism, senescence, and proliferation [92]. However, within the pathological microenvironment of diabetic bone defects, hyperglycemia‐induced mitochondrial dysfunction manifests as ETC dysregulation, the excessive leakage of free electrons, and consequent ROS overproduction [60]. This pathological milieu concurrently impairs the enzymatic activities of catalase (CAT) and superoxide dismutase (SOD), establishing an intense cycle of ROS accumulation and progressive mitochondrial deterioration [93]. Current therapeutic strategies for diabetic bone defects predominantly focus on ROS scavenging while overlooking the pivotal regulatory role of mitochondrial homeostasis. In this study, we developed the MF@Y nanozyme that not only effectively scavenges ROS but also, more importantly, exerts systemic protection through the regulation of mitochondrial metabolism. Our GSEA results revealed the significant activation of mitochondria‐associated pathways, including superoxide metabolism, energy homeostasis, and the oxidative stress response, following MF@Y treatment. Comprehensive evaluations using the MitoSOX Green probe, biological TEM, and JC‐10 staining confirmed the capacity of MF@Y to maintain mitochondrial structural integrity and function under high‐ROS conditions. Building upon established mechanisms wherein mtROS accumulation induces mitochondrial membrane depolarization and ATP synthesis impairment, excessive levels of mtROS trigger mPTP opening, facilitating mtDNA cytoplasmic translocation as damage‐associated molecular patterns (DAMPs) to activate the cGAS‒STING pathway [52, 94]. We further revealed the ability of MF@Y to suppress mPTP overactivation and prevent mtDNA leakage. Reactome analysis revealed that the MF@Y nanozyme upregulated genes related to interferon α/β signaling, the response to type I interferon, and interferon type I signaling pathways, indicating the crucial effects of the MF@Y nanozyme on type I interferon‐related pathways. Multiple studies have shown that activation of the cGAS‒STING pathway is critical for the secretion of type I interferons [53, 95]. Consequently, we hypothesized that the MF@Y nanozyme can suppress cGAS‒STING pathway activation triggered by cytoplasmic mtDNA, thereby reducing the release of type I interferons. Experimental validation using TBHP‐induced models combined with p‐STING immunofluorescence and RT‐qPCR (*IFNA1*, *IFNB1*, and *IFNA2*) analyses confirmed the efficacy of MF@Y in suppressing STING phosphorylation and downregulating interferon expression. Collectively, our findings elucidate a mechanistic cascade whereby the MF@Y nanozyme scavenges mtROS, preserves the ΔΨm, regulates mPTP dynamics, prevents mtDNA cytoplasmic translocation, and inhibits cGAS‒STING pathway activation, providing a novel therapeutic paradigm for diabetic bone defects.

Cellular senescence is a pivotal pathological driver of impaired diabetic bone regeneration and is characterized by the compromised osteogenic differentiation of BMSCs and dysfunctional vascular remodeling [96, 97, 98]. Our KEGG and Reactome analyses revealed significant modulation of senescence‐related pathways, particularly the longevity‐regulating pathway, AMPK signaling, and the SASP network, by the MF@Y nanozyme. Subsequent experiments demonstrated that the MF@Y nanozyme effectively counteracts cellular senescence through three principal mechanisms. First, it suppresses SA‐β‐gal activity by downregulating key senescence markers (*p21* and *p16*) and SASP factors (*IL‐6*, *CXCL1*, *CXCL10*, and *MMP1*). Second, combinatorial assessment using 𝛾‐H2AX and EdU incorporation assays revealed significant mitigation of the DNA damage response and restoration of the proliferative capacity. Third, TEM ultrastructural analysis coupled with ΔΨm measurements (JC‐10 staining) revealed preserved mitochondrial integrity and function in the nanozyme‐treated groups. The mitochondrial protection mechanism emerged as a central therapeutic axis and was supported by experimental evidence including the prevention of TBHP‐induced cristae disorganization and matrix swelling observed via TEM, the reduction in mtROS accumulation compared with that in the TBHP‐treated group, the suppression of pathological mPTP opening, and the decrease in cytoplasmic mtDNA leakage. Our data suggest that the MF@Y nanozyme intercepts this cascade at its origin by maintaining the mitochondrial redox balance and preserving genomic stability, thereby preventing both replicative and stress‐induced senescence phenotypes.

This study indicates that Yoda1, a selective Piezo1 agonist, exerts no significant effect on macrophage M1 polarization whilst modestly promoting M2 polarization, which illuminates the subtype‐specific role of Piezo1 in regulating macrophage functional plasticity. Regarding M2 polarization, Atcha et al. showed that Piezo1 knockout or pharmacological inhibition markedly attenuated IL‐4‐induced M2 marker expression, whereas Yoda1‐mediated Piezo1 activation further enhances this effect [99]. Mechanistically, Piezo1 activation drives Ca^2+^ influx that engages the phosphatase calcineurin, leading to activation and nuclear translocation of NFAT. Once in the nucleus, NFAT can either form a transcriptional complex with STAT6, the master regulator of M2 polarization, or bind directly to the promoter regions of M2 signature genes, thereby synergistically amplifying IL‐4/IL‐13‐driven STAT6 signaling. Additionally, Ca^2+^ signaling may additionaly promote M2 polarization through metabolic reprogramming linked to mitochondrial oxidative phosphorylation, although this regulatory axis awaits further experimental validation [100]. In contrast, existing evidence supports the view that Piezo1 is not an obligatory regulator of M1 polarization. Atcha et al. reported that Piezo1 deficiency or inhibition had no discernible effect on LPS/IFN‐γ‐induced M1 marker expression [99]. Multiple studies confirm Yoda1 alone fails to induce M1 polarization and can suppress pro‐inflammatory cytokine release in some contexts [101, 102]. Three principal mechanisms account for this phenotypic divergence. M1 polarization is primarily orchestrated by TLR4/IFNγR‐NF‐κB/STAT1 cascades, in which Piezo1‐mediated Ca^2+^ signals function as a secondary modulator and are therefore insufficient to meaningfully amplify an already potent pro‐inflammatory program. Furthermore, Ca^2+^ influx can activate phosphatases (e.g., calcineurin) or induce anti‐inflammatory factors, potentially counteracting pro‐inflammatory pathways and limiting net effects on M1 polarization. In addition, LPS/IFN‐γ constitutes a powerful polarizing stimulus, and the magnitude of Yoda1‐driven Ca^2+^ influx is unlikely to reach the threshold required to augment it; conversely, IL‐4‐mediated M2 signaling is comparatively modest in amplitude and thus more susceptible to cooperative enhancement by Piezo1 activity. Collectively, the observation that Yoda1 selectively promotes M2 polarization whilst exerting minimal influence on M1 polarization suggests that Piezo1 engagement selectively skews macrophages towards an anti‐inflammatory, reparative phenotype, thereby facilitating inflammation resolution and tissue repair. The apparent insensitivity of M1 polarization to Piezo1 activity represents a biological safeguard, ensuring that incidental mechanical stimuli do not inadvertently trigger aberrant inflammatory cascades.

Therapeutic modulation of bone metabolic homeostasis represents a pivotal yet underexplored strategy for addressing impaired bone regeneration in diabetic patients [103]. While physiological bone remodeling relies on a tightly orchestrated equilibrium between osteoblastic bone formation and osteoclastic bone resorption [104], diabetic bone defects exhibit pathologically skewed dynamics characterized by defective osteogenesis and exacerbated osteoclastogenesis [14]. Our findings reveal that mitochondrial dysfunction‐induced ROS overproduction serves as a critical nexus linking hyperglycemia to impaired BMSC functionality. Recent diabetic bone studies have suggested that excessive ROS accumulation not only induces BMSC senescence and diminishes multipotency but also reprograms differentiation fate by activating PPARγ‐mediated adipogenesis while suppressing RUNX2‐driven osteogenesis [105, 106]. Concurrently, ROS hyperactivation amplifies osteoclastogenesis by stimulating the RANKL/NFATc1 pathway, creating a self‐perpetuating cycle of bone loss [105]. To disrupt this cycle, we engineered the MF@Y nanozyme that concurrently addresses oxidative stress and mechanotransduction deficits. By localizing to mitochondria in BMSCs, the MF@Y nanozyme scavenges ROS, effectively reversing the SASP and proinflammatory cytokine cascades. This restoration of redox homeostasis rejuvenates the migratory capacity of BMSCs and redirects differentiation commitment from adipocytes to functional osteoblasts, as evidenced by the upregulation of osteogenesis‐related genes and enhanced mineralization. Notably, the Yoda1 component activates Piezo1‐mediated calcium signaling, which synergistically enhances Wnt/β‐catenin osteogenic programming. Similarly, the MF@Y nanozyme suppresses osteoclasts, reducing TRAP‐positive multinucleated cell formation and resorptive activity. The therapeutic translation potential of the BCPG‐MF@Y scaffold was substantiated by micro‐CT analyses, which revealed that, compared with other groups, the BCPG‐MF@Y scaffold enhanced the bone volume fraction in the defect site through coordinated anabolic/catabolic regulation. This bidirectional modulation surpasses current monotherapy approaches focused solely on osteoblast stimulation, positioning the MF@Y nanozyme as a paradigm‐shifting platform for metabolic bone disorders.

This study identifies MF‐mediated mitochondrial protection and Yoda1‐mediated Piezo1 channel activation as the two core mechanisms for MF@Y. Whether these two mechanisms engage in crosstalk or mutually reinforcing interactions represents a question of considerable scientific interest. Integrating our findings with the existing literature, we propose that these mechanisms may operate synergistically and potentially establish a positive feedback loop, although causal relationships between them have not been directly demonstrated in the present study. Mitochondrial functional status can influence Piezo1 activity through several interconnected mechanisms. Our data demonstrate that MF effectively scavenges excess ROS whilst preserving ΔΨm and ATP synthesis. As the principal intracellular source of ROS, dysfunctional mitochondria precipitate redox imbalance with broad downstream consequences. Notably, mechanosensitive channels including Piezo1 exhibit marked sensitivity to the redox environment; ROS accumulation has been shown to suppress channel activity and alter gating kinetics [107, 108]. By sustaining mitochondrial homeostasis, MF may therefore optimize the functional microenvironment of Piezo1, enhancing its responsiveness to mechanical stimuli and to Yoda1. Reciprocally, Piezo1 activation modulates mitochondrial function. Yoda1‐mediated Piezo1 activation elicited robust Ca^2+^ influx, and at physiologically appropriate concentrations, Ca^2+^, as an essential second messenger, promotes tricarboxylic acid cycle activity and mitochondrial ATP synthesis whilst engaging cytoprotective signaling cascades including CREB and Akt [33, 109]. The observation that Yoda1 alone was sufficient to promote angiogenesis and osteogenic differentiation further implies that it may augment cellular energy metabolism through Ca^2+^‐dependent signalling. In this study, within the context of oxidative stress attenuation and mPTP stabilization afforded by MF, Yoda1‐induced Ca^2+^ influx may synergistically reinforce mitochondrial function, establishing an auto‐amplifying energy signaling cycle. Collectively, we propose that the interaction between MF and Yoda1 within MF@Y does not represent a simple additive superposition of independent effects, but rather a coordinated mechanistic cascade: MF preserves mitochondrial integrity, thereby optimizing the functional microenvironment of Piezo1 and enabling efficient Yoda1‐mediated channel activation. The resulting calibrated Ca^2+^ signal in turn promotes mitochondrial bioenergetics and cytoprotective signaling, further amplifying downstream cellular responses, including angiogenesis and osteogenic differentiation. This mechanistic coupling most significantly accounts for why MF@Y substantially outperforms either constituent in isolation, exceeding what simple additivity would predict. In addition, it should be noted that the present study focuses primarily on validating the overall efficacy of MF@Y and characterizing the independent contributions of its two core components, without delineating the precise molecular interface between these pathways, including whether MF directly regulates Piezo1 expression or subcellular localization, and the specific mechanisms by which Ca^2+^ signaling dynamics downstream of Piezo1 govern mitochondrial bioenergetics. These unresolved questions represent both an inherent limitation of the current work and a compelling direction for future investigation.

In terms of clinical translation, the scaffold's core components have favorable safety profiles. The 3D printing platform enables personalized fabrication for precise defect matching. The MF nanozyme component, HFn, is produced by recombination, facilitating scalable manufacturing. The scaffold targets refractory indications including periodontitis, postoperative jaw bone defects, peri‐implantitis bone defects, and osteoporotic or traumatic bone defects in the limbs, offering a novel regenerative therapeutic approach for diabetes‐related bone defects. Mechanistically, this work establishes a multi‐pathway synergistic regulatory paradigm, providing new theoretical insights for diabetic bone regeneration.

## 4 Conclusion

In summary, we report a diamond cubic lattice BCPG‐MF@Y scaffold that coordinates mitochondrial homeostasis and mechanotransduction to reprogram the osteoimmune microenvironment for diabetic bone regeneration. By simultaneously targeting excessive mtROS, cellular senescence, and chronic inflammation, the Yoda1‐functionalized biomimetic nanozymes rescue mitochondrial dysfunction, direct macrophage polarisation towards a pro‐regenerative phenotype, bidirectionally regulate BMSCs differentiation, suppress osteoclastogenesis, and ultimately enable vascularised bone regeneration that mono‐targeted approaches have failed to achieve. Translationally, this scaffold holds immediate clinical relevance for critical‐sized craniofacial and long‐bone defect reconstruction in diabetic patients. Its potential applications extend to deployment as a bioactive interfacial coating for titanium or PEEK implants to enhance osseointegration in metabolically compromised hosts, and as a 3D‐printable platform adaptable to spinal fusion and maxillofacial reconstruction. Beyond orthopedic indications, our dual‐targeted strategy that synergizes mitochondrial rehabilitation with mechano‐bioactivation establishes a pioneering therapeutic paradigm for chronic inflammatory disorders with mitochondrial pathophysiology, including osteoarthritis and senescent tissue degeneration.

## 5 Experimental Section

### 5.1 Chemicals and Reagents

The MF nanozymes were purchased from Xi'an Qiyue Biology (Xi'an, China). Yoda1 (T7506), molecular weight 355.27 g/mol, was purchased from TargetMol (Wellesley Hills, MA, USA), with a purity of ≥99.98%. pGelMA (EFL‐GM‐PR‐002) and alkene‐coupled hydrogel fluorescent dyes (EFL‐DYE‐UF‐ENE‐R) were obtained from Engineering For Life (Suzhou, China). The Cy7‐labeled pGelMA was purchased from Suzhou Xianjue New Materials Technology Co. Ltd (Suzhou, China). The BCP ceramic scaffolds (20% hydroxyapatite and 80% β‐tricalcium phosphate) were procured from Ten Dimensions (Guangdong) Technology Co. Ltd. (Guangdong, China). TBHP was purchased from Macklin (Shanghai, China). Fluorescein 5‐isothiocyanate (FITC; F8070), DAPI (C0060), and the DCFH‐DA assay kit (CA1410) were obtained from Solarbio (Beijing, China). PK Mito Orange (PKMO) was purchased from Genvivo (Nanjing, China). SYBR Gold (S11494) was procured from Thermo Fisher Scientific (Waltham, MA, USA). Calcein‐AM (C1367) and Hoechst 33342 (C1029) were purchased from Beyotime (Shanghai, China). Streptozotocin (STZ; CS10491) was purchased from Coolaber Science & Technology (Beijing, China).

### 5.2 Characterization of the MF Nanozymes

First, a transmission electron microscope (JEM‐F200, Jeol Ltd., Tokyo, Japan) was used to analyze the morphology of the MF nanozyme and obtain EDS spectra. Following previous methods [13, 110], FTIR spectroscopy (Nicolet 6700, Thermo Fisher Scientific) was used to analyze the structure of the MF nanozyme. Its size was measured with a DLS analyzer (DelsaMax Pro, Beckman Coulter Life Sciences, Brea, CA, USA). EPR was used to evaluate the ability of the MF nanozyme to scavenge •OH and O_2_^•−^ radicals. These radicals were generated upon xenon lamp irradiation in the presence of 50 mM H_2_O_2_, and 5,5‐dimethyl‐1‐pyrroline‐N‐oxide (DMPO; 100 mM) was employed in aqueous solution to capture •OH and in methanol solution to capture O_2_^•−^. A Bruker EPR EMX Plus spectrometer (Karlsruhe, Germany) was used to monitor the EPR signal after the MF nanozyme was administered at 0, 10, or 20 µg/mL for radical scavenging.

### 5.3 Characterization of the BCP Scaffold

Micro‐CT (Bruker Skyscan 1276, Bruker Corp., Billerica, MA, USA) was used to evaluate the external morphology and internal architecture of the BCP scaffold, and CTvox software was used for data analysis. The surface and cross‐sectional morphologies of the BCP scaffold were analyzed with a scanning electron microscope (Hitachi Regulus 8100, Hitachi, Tokyo, Japan).

### 5.4 Characterization of the pGelMA Hydrogel

pGelMA was dissolved in a 0.25% (w/v) lithium phenyl‐2,4,6‐trimethylbenzoylphosphinate (LAP) photoinitiator solution to yield a 6% (w/v) pGelMA precursor. The pGelMA hydrogel was generated upon exposure to 405 nm ultraviolet light. The hydrogel was subsequently freeze‐dried, cut into small pieces, and gilded, and cross‐sectional images were captured using a scanning electron microscope (JCM‐7000, Jeol Ltd.). pGelMA‐ and alkene‐coupled hydrogel fluorescent dyes were subsequently dissolved in 0.25% (w/v) LAP photoinitiator solution. Following exposure to 405 nm ultraviolet light, the pGelMA hydrogel was observed using a confocal microscope (C2+, Nikon, Tokyo, Japan).

### 5.5 Preparation and Characterization of the MF@Y Nanozymes

First, Yoda1 was dissolved in dimethyl sulfoxide (DMSO), and this solution was mixed with the MF nanozyme solution. After stirring for 48 h, the obtained solution was centrifuged for 10 min at 12 000 × *g* and washed three times with deionized water to obtain the MF@Y nanozyme. An X‐ray photoelectron spectrometer (Thermo Scientific K‐Alpha, Thermo Fisher Scientific) was used to collect the XPS spectra of Yoda1, the MF nanozyme, and the MF@Y nanozyme.

### 5.6 Preparation of the BCPG, BCPG‐MF, and BCPG‐MF@Y Scaffolds

The MF and MF@Y nanozymes were incorporated into the 6% (w/v) pGelMA precursor to produce pGelMA‐MF (at a final MF nanozyme concentration of 50 µg/mL) and pGelMA‐MF@Y precursors, respectively. The MF@Y nanozyme in the pGelMA‐MF@Y precursor contained 50 µg/mL MF nanozyme with full Yoda1 adsorption. The BCP scaffold was subsequently immersed in the pGelMA, pGelMA‐MF, or pGelMA‐MF@Y precursor, and the coated BCP scaffolds were then exposed to 405 nm ultraviolet light to yield the BCPG, BCPG‐MF, and BCPG‐MF@Y scaffolds, respectively.

### 5.7 Characterization of the BCPG‐MF@Y scaffold

Building on our previously established protocols [65], MF‐FITC nanoparticles were fabricated and subsequently incorporated into the BCPG‐MF@Y scaffold. To characterize the degradation kinetics and cargo release profile of the scaffold, samples were immersed in PBS supplemented with type I collagenase (1 U/mL, Share‐bio, Shanghai, China). At predetermined time intervals, scaffold mass was recorded, and supernatants were collected and subjected to multi‐parametric analysis, including quantification of MF‐FITC fluorescence intensity, Yoda1 concentration, pH, and zeta potential.

### 5.8 Molecular Docking

A molecular docking study was performed to investigate the binding mode between Yoda1 and one of the subunits of HFn using AutoDock Vina 1.1.2 [111]. The 3D structure of the HFn subunit (PDB ID: 6B8F) was downloaded from the RCSB Protein Data Bank (www.rcsb.org). The 2D structure of Yoda1 was constructed with ChemBioDraw Ultra 14.0, and the 2D structure was converted to a 3D structure with ChemBio3D Ultra 14.0. The AutoDockTools 1.5.6 package [112, 113] was employed to generate the docking input files. The ligand was prepared for docking by merging nonpolar hydrogen atoms and defining rotatable bonds. The search grid for ferritin was defined as center_x: 9.897, center_y: 39.248, and center_z: 39.248 with dimensions of size_x: 47.25, size_y: 47.25, and size_z: 47.25. To increase docking accuracy, the exhaustiveness value was set to 16. In addition, the num_modes value was set to 30. The default docking parameters were used if not otherwise mentioned. Eleven docking poses were selected, and then molecular dynamics analysis was performed to revise the docking results.

### 5.9 Molecular Dynamics Simulations

Amber 14 [111, 114, 115] and AmberTools 15 were used for molecular dynamics simulations of the selected docked poses. The Yoda1 poses were first prepared by ACPYPE [116], a tool based on ANTECHAMBER [116, 117], to generate automatic topologies and parameters in different formats for different molecular mechanics programs, including the calculation of partial charges. The leaprc.gaff forcefield was subsequently used to prepare the ligands, whereas leaprc.ff12SB was used for the receptor. The system was placed in a rectangular box (with a 10.0 Å boundary) of TIP3P water using the SolvateOct command, with the minimum distance between solute atoms. Equilibration of the solvated complex was performed via short minimization (500 steps of each steepest descent and conjugate gradient method), 500 ps of heating, and 50 ps of density equilibration with weak restraints using the GPU (NVIDIA Tesla K20c)‐accelerated PMEMD module. Finally, 40 ns of molecular dynamics simulations were carried out.

### 5.10 Cell Culture

HMEC‐1 human microvascular endothelial cells were acquired from ATCC (Manassas, VA, USA), and HBMSCs were acquired from Cyagen Biosciences (Guangzhou) Inc. (Guangzhou, China). The cells were cultured in complete culture medium composed of DMEM (high glucose, 4.5g/L, Gibco, Thermo Fisher Scientific), 10% fetal bovine serum (FBS; Cyagen Biosciences), and 1% penicillin–streptomycin–amphotericin B solution (Beyotime) under 5% CO_2_ at 37°C.

### 5.11 Isolation and Culture of Bone Marrow‐Derived Macrophages (BMMs)

As described in previous studies [118, 119], the femurs and tibias of 4‐week‐old mice were isolated and collected after sacrifice. Then, culture medium (α‐MEM) was used to flush the bone marrow cavities. The bone marrow cells were cultured with complete culture medium supplemented with 20 ng/mL M‐CSF (Novoprotein, Suzhou, China) to obtain BMMs. The resulting BMMs were subsequently used for macrophage polarization, osteoclastic differentiation, and bone resorption assays.

### 5.12 RNA Isolation, Library Preparation, RNA‐seq and Data Analysis

As described in a previous study [65], total RNA was extracted with TRIzol reagent (Thermo Fisher Scientific). The RNA purity and concentration were measured with a NanoDrop 2000 spectrophotometer (Thermo Fisher Scientific), and RNA integrity was assessed using an Agilent 2100 bioanalyzer (Agilent Technologies, Santa Clara, CA, USA). The VAHTS Universal V6 RNA‐seq Library Prep Kit was subsequently used to construct libraries. OE Biotech Co., Ltd. (Shanghai, China) assisted in transcriptome sequencing and analysis. The procedures employed for RNA‐seq and data analysis in this study were based on those in our prior publications [65, 120].

### 5.13 Fluo‐4 AM Assay

As described in a previous study [121], HBMSCs were seeded into 24‐well plates (Jet Bio‐Filtration Co. Ltd., Guangzhou, China) at a density of 5 × 10^4^ cells/well. The next day, the cells were treated with the MF nanozyme (10 µg/mL), Yoda1 (0.4 µM), or the MF@Y nanozyme (10 µg/mL MF nanozyme with sufficient adsorption of Yoda1) for 4 h. Then, the cells were washed with Hanks balanced salt solution (HBSS) and incubated with Fluo‐4 AM (F8500, Solarbio) according to the manufacturer's protocol. After the samples were washed with HBSS, fluorescence images were captured using a microscope (Eclipse Ts2R‐FL, Nikon).

### 5.14 Organelle‐Level Dynamic Trafficking

As described in previous studies [122, 123], HBMSCs were seeded into laser confocal dishes (NEST Biotechnology Co. Ltd, Wuxi, China) at a density of 2 × 10^5^ cells/well. The next day, the cells were treated with the MF‐FITC@Y nanozyme. At predetermined time intervals, a confocal laser scanning microscope (C2+, Nikon) was used to assess the dynamic transport pathway of MF@Y. An anti‐LAMP1 antibody (9091, Cell Signaling Technology, Danvers, MA, USA), an anti‐Rab5 antibody (3547, Cell Signaling Technology), an anti‐TOM20 antibody (42406, Cell Signaling Technology), and a CoraLite594‐conjugated Goat Anti‐Rabbit IgG(H+L) (SA00013‐4, Proteintech, Rosemont, IL, USA) were used for immunofluorescence staining.

### 5.15 DCFH‐DA Assay

As described in previous studies [120], HBMSCs were seeded into 24‐well plates at a density of 5 × 10^4^ cells/well. The next day, the cells were incubated with the MF nanozyme, Yoda1, or the MF@Y nanozyme for 2 h and treated with TBHP (100 µM). Two hours later, the cells were washed with phosphate‐buffered saline (PBS) and incubated with DCFH‐DA and Hoechst 33342. Finally, fluorescence images were captured using a microscope (Eclipse Ts2R‐FL, Nikon).

### 5.16 Morphological Analysis of Mitochondria by Bio‐TEM

HBMSCs were seeded into 6‐well plates at a density of 2 × 10^5^ cells/well. When the cells reached 70% confluence, they were treated with the MF nanozyme, Yoda1, or the MF@Y nanozyme for 2 h and then treated with TBHP for 2 h. Next, the cells were collected and fixed with 2.5% glutaraldehyde at 4°C. After dehydration, resin penetration, embedding, polymerization, ultrathin section staining, and staining with uranyl acetate, TEM (HT7800, Hitachi) was used to observe mitochondrial structure and morphology.

### 5.17 mtROS Detection

The superoxide indicator MitoSOX Green (M36005; Thermo Fisher Scientific) was used to detect mtROS. HBMSCs were seeded into 24‐well plates at a density of 5 × 10^4^ cells/well. The next day, the cells were treated with the MF@Y nanozyme for 2 h and then with TBHP (100 µM); untreated cells were used as controls. After an additional 2 h, the cells were washed with PBS and incubated with MitoSOX Green, PKMO, and Hoechst 33342 for 30 min. Fluorescence images were captured using a confocal laser scanning microscope (C2+, Nikon).

### 5.18 mtDNA Detection

SYBR Gold and PKMO were used to label mtDNA and mitochondria, respectively. HBMSCs were seeded into laser confocal dishes (NEST Biotechnology Co. Ltd.) at a density of 2 × 10^5^ cells/well. The next day, the cells were treated with the MF@Y nanozyme for 2 h and then with TBHP. After an additional 2 h, the cells were washed with PBS and incubated with SYBR^TM^ Gold and PKMO for 20 min. Fluorescence images were captured with a DeltaVision OMX SR (Cytiva, Marlborough, MA, USA).

### 5.19 Measurement of the ΔΨm

A JC‐10 kit (CA1310, Solarbio) was used to measure the ΔΨm. As described in previous studies [120, 124], HBMSCs were seeded into 24‐well plates at a density of 5 × 10^4^ cells/well. The next day, the cells were treated with the MF nanozyme, Yoda1, or the MF@Y nanozyme for 2 h and then with TBHP. After an additional 2 h, the cells were washed with PBS and incubated with JC‐10 for 20 min. Following washing with JC‐10 buffer at 4°C, fluorescence images were captured using a DMi8 microscope (Leica, Wetzlar, Germany).

### 5.20 Measurement of ATP Content

HBMSCs were seeded into 96‐well plates at a density of 5 × 10^3^ cells per well. The following day, cells were pretreated with MF@Y nanozymes for 2 h prior to TBHP stimulation. Intracellular ATP content was subsequently quantified using an ATP Assay Kit (AKOP004M, Beijing Boxbio Science & Technology Co. Ltd., Beijing, China) according to the manufacturer's instructions.

### 5.21 Assessment of mPTP Opening

As described in previous studies [125, 126], HBMSCs were seeded into 24‐well plates at a density of 5 × 10^4^ cells/well. The next day, the cells were treated with the MF@Y nanozyme for 2 h and then with TBHP. An mPTP assay kit (C2009S, Beyotime) was then used to examine the opening of the mPTP. After washing the cells with PBS three times, fluorescence images were captured using a confocal laser scanning microscope (C2+, Nikon).

### 5.22 Transwell Assay

As described in previous studies [10, 127], 1 × 10^5^ cells were seeded into the upper chamber of each well of a Transwell plate, and complete medium containing TBHP, TBHP+MF, TBHP+Yoda1, or TBHP+MF@Y was added to the lower chambers. After 10 h, 0.5% crystal violet was used to stain the cells in the upper chambers. Then, PBS was used to wash away excess dye. Next, the cells that had not penetrated the Transwell membranes were gently removed. Finally, a Nikon microscope (Eclipse Ts2R‐FL) was used to capture images.

### 5.23 Tube Formation Assay

As described in a previous study [120], 10 µL of Matrigel (BD Biosciences, Franklin Lakes, NJ, USA) was added to each well of a 24‐well plate and spread evenly. After incubation at 37°C for 1 h, HMEC‐1 cells were seeded into the wells at a density of 1.5 × 10^5^ cells/well and cultured with complete medium containing TBHP, TBHP+MF, TBHP+Yoda1, or TBHP+MF@Y. Six hours later, calcein‐AM was used to stain the cells for 30 min. Finally, a Nikon microscope (Eclipse Ts2R‐FL) was used to capture fluorescence images.

### 5.24 SA‐β‐gal Staining

In accordance with previous studies [97], HBMSCs were seeded into 6‐well plates at a density of 2 × 10^5^ cells/well. The next day, the cells were treated with the MF nanozyme, Yoda1, or the MF@Y nanozyme for 2 h and then with TBHP. The cells were subsequently stained with a SA‐β‐gal staining kit (G1580, Solarbio) according to the manufacturer's protocol. A Nikon microscope (ECLIPSE Ts2R‐FL) was used to capture fluorescence images.

### 5.25 EdU Staining

As described in previous studies [128, 129], HBMSCs were seeded into 24‐well plates at a density of 5 × 10^4^ cells/well. The next day, the cells were treated with the MF nanozyme, Yoda1, or the MF@Y nanozyme for 2 h and then with TBHP. A Cell‐Light EdU Apollo 488 In Vitro Kit (C10310‐3, Guangzhou RiboBio Co. Ltd., Guangzhou, China) was used to assess cell proliferation. Fluorescence images were captured using a Leica DMi8 microscope.

### 5.26 γ‐H2AX Staining

As described in previous studies [65, 130], HBMSCs were seeded into 24‐well plates at a density of 5 × 10^4^ cells/well. The next day, the cells were treated with the MF nanozyme, Yoda1, or the MF@Y nanozyme for 2 h and then with TBHP. A γ‐H2AX immunofluorescence staining kit (C2035S, Beyotime) was then used to detect DNA damage. Fluorescence images were captured using a Leica DMi8 microscope.

### 5.27 P‐STING Staining

As described in previous studies [53, 131], HBMSCs were seeded into 24‐well plates at a density of 5 × 10^4^ cells/well. The next day, the cells were treated with the MF@Y nanozyme for 2 h and then with TBHP. After fixing with 4% paraformaldehyde (PFA), the cells were incubated with an anti‐p‐STING antibody (HA722832, HUABIO, Hangzhou, China) and anti‐rabbit IgG (H+L), F(ab’)_2_ fragment (Alexa Fluor 488‐conjugated) (4412, Cell Signaling Technology), and immunofluorescence images were captured with a confocal laser scanning microscope (C2+, Nikon).

### 5.28 Osteogenic Differentiation of HBMSCs

As previously described [65, 132], HBMSCs were seeded into 24‐well plates at a density of 5 × 10^4^ cells/well. When the cells reached 70% confluence, they were cultured with osteogenic differentiation medium (HUXMX‐90021, Cyagen Biosciences) containing TBHP (25 µM), TBHP+MF, TBHP+Yoda1, or TBHP+MF@Y. After 14 days, ALP activity and calcium mineral deposition were evaluated via an ALP assay kit (C3206, Beyotime) and ARS (Cyagen Biosciences) staining, respectively. Finally, images were captured with a camera (DSC‐RX1, Sony, Tokyo, Japan) and microscope (Eclipse Ts2R‐FL).

### 5.29 Adipogenic Differentiation of HBMSCs

HBMSCs were seeded into 24‐well plates at a density of 5 × 10^4^ cells/well. When the cells reached 100% confluence, they were cultured with adipogenic differentiation medium (HUXMX‐90031, Cyagen Biosciences) containing TBHP (25 µM), TBHP and the MF nanozyme, TBHP and Yoda1, or TBHP and the MF@Y nanozyme. After 21 days, Oil Red O staining was used to assess the adipogenic differentiation of the HBMSCs. Images were captured with a Nikon microscope (Eclipse Ts2R‐FL).

### 5.30 Macrophage Polarization

In accordance with previous studies [133, 134], BMMs were seeded into 6‐well plates, and lipopolysaccharide (LPS; 500 ng/mL, L2630, Merck KGaA) was used to induce M1 polarization. IL‐4 (40 ng/mL, CK15, Novoprotein) and IL‐13 (40 ng/mL, CH18, Novoprotein) were used to induce M2 polarization. In addition, TBHP, TBHP, and the MF nanozyme, TBHP and Yoda1, or TBHP and the MF@Y nanozyme were added to the culture medium. After 24 h, a flow cytometer (CytoFLEX, Beckman Coulter Life Sciences) and a Leica DMi8 microscope were used to assess macrophage polarization. An anti‐iNOS antibody (bs‐0162R, Bioss, Beijing, China), an anti‐mannose receptor (CD206) antibody (ab64693, Abcam, Cambridge, MA, USA), a rhodamine (TRITC)‐conjugated AffiniPure anti‐rabbit IgG (H+L) (700025152, Jackson ImmunoResearch, Newmarket, UK), and an anti‐rabbit IgG (H+L), F(ab’)_2_ fragment (Alexa Fluor 488‐conjugated) (4412, Cell Signaling Technology) were used for immunofluorescence staining. Additionally, an anti‐F4/80 antibody (565411, BD Pharmingen, Franklin Lakes, NJ, USA), an anti‐CD86 antibody (560582, BD Pharmingen), and an anti‐CD206 antibody (565250, BD Pharmingen) were used for flow cytometry.

### 5.31 Osteoclastic Differentiation Assay

As described in previous studies [135], BMMs were seeded into 6‐well plates and cultured using an Osteoclast Culture Kit (AMK1001, Hangzhou Yangming Biotechnology Co. Ltd., China) to induce osteoclastic differentiation. In addition, TBHP, TBHP and the MF nanozyme, TBHP and Yoda1, or TBHP and the MF@Y nanozyme were added to the culture medium. After 7 days, the cells were fixed with 4% PFA and multinucleated osteoclasts were stained with a TRAP staining kit (387A, Merck KGaA) and TRITC‐labelled phalloidin (40734ES75, Yeasen, Shanghai, China) according to the manufacturer's instructions. Finally, images were captured with a camera (DSC‐RX1, Sony) and microscope (Eclipse Ts2R‐FL, Nikon).

### 5.32 Bone Resorption Assay

In accordance with previous studies [65, 136], BMMs were seeded onto bovine bone discs (DT‐1BON1000‐96, Immunodiagnostic Systems Holdings Ltd., Tyne & Wear, UK) and cultured with osteoclastic differentiation medium containing TBHP, TBHP+MF, TBHP+Yoda1, or TBHP+MF@Y for 14 days. Then, 1% ammonia water was used to remove the cells attached to the bovine bone discs. After the discs were dehydrated, dried, and sprayed with gold, a scanning electron microscope (JCM‐7000, Jeol Ltd.) was used to observe bone resorption lacunae.

### 5.33 Live/Dead Assay

As described in previous studies [14, 68], the Live/Dead assay was used to examine the biocompatibility of different hydrogels. HBMSCs were mixed with a 6% pGelMA hydrogel containing the MF nanozyme, Yoda1, or the MF@Y nanozyme, and 200 µL of each hydrogel precursor was added to each well of a 24‐well plate. After irradiation with 405 nm ultraviolet light and culture for 3 or 5 days, the cells were stained with calcein‐AM and PI for 1 h. After washing the cells with PBS, images were captured using a confocal laser scanning microscope (C2+, Nikon).

### 5.34 Cell Adhesion Assay

As described in a previous study [66], HBMSCs were seeded onto the surface of the BCP, BCPG, BCPG‐MF, or BCPG‐MF@Y scaffold. After 5 days, the cells were fixed with PFA and stained with TRITC‐labelled phalloidin and DAPI. Finally, images were captured using a confocal laser scanning microscope (C2+, Nikon).

### 5.35 RT‐qPCR

The procedures employed for RT‐qPCR in this study are detailed in our prior publications [137, 138, 139]. The primers used were provided by BioTNT (Shanghai, China), and their sequences are listed in Table S2. PCR kits were purchased from Yeasen (11204ES08).

### 5.36 Animal Experiments

Ethical approval was granted by the Ethics Committee of Shanghai Sixth People's Hospital Affiliated to Shanghai Jiao Tong University School of Medicine (approval number: 2021‐0767). All experimental procedures were approved by the Animal Care and Experiment Committee of Shanghai Sixth People's Hospital Affiliated to Shanghai Jiao Tong University School of Medicine. In accordance with the methods of previous studies [64, 140], male Sprague‐Dawley (SD) rats (eight weeks old) were injected intraperitoneally with 1% STZ (65 mg/kg) dissolved in 0.1 M citrate buffer (pH 4.5). When the fasting blood glucose of the rats was greater than 16.7 mmol/L for two consecutive days, the diabetic rat model was considered successfully established. The rats were then randomly divided into five groups: control, BCP, BCPG, BCPG‐MF, and BCPG‐MF@Y. Next, the rats were anesthetized by intraperitoneal injection of 5% pentobarbital sodium, and an annular bit was used to create 5‐mm cranial bone defects. After implantation of the different scaffolds at the defect sites, the skin was sutured. Finally, at the indicated times, the rats were sacrificed, and the skulls were collected and fixed in formalin.

A BCPG‐Yoda1 group was excluded from the experimental design on the basis of Yoda1's physicochemical incompatibility with aqueous hydrogel matrices. Being a hydrophobic compound, Yoda1 undergoes aggregation and precipitation rather than uniform dispersion upon direct admixture into water‐based hydrogel systems, precluding stable and reproducible drug loading. Furthermore, such formulations are characterized by a pronounced burst‐release profile, typically liberating more than 50%, and in many instances upward of 80%, of the total drug payload within the first 24 h, rendering sustained therapeutic concentrations at the implantation site unattainable [141, 142]. This precipitous drug loss not only abrogates prolonged local efficacy but also risks generating transiently supraphysiological concentrations with attendant cytotoxic consequences.

### 5.37 Animal In Vivo Imaging

The BCPG‐MF@Y scaffolds were constructed using Cy7‐labeled pGelMA hydrogel, and implanted into the cranial defect sites of diabetic rats. At designated time points, an in vivo fluorescence and bioluminescence imaging system (VISQUE InVivo ART 100, VIEWORKS Co. Ltd., Gyeonggi‐do, Republic of Korea) was used to detect the degradation of the pGelMA hydrogel within the scaffold.

### 5.38 Vascular Perfusion and Micro‐CT Analysis

Assessment of neovascularization: at 7 weeks after surgery, the rats were euthanized, and heparinized saline, PFA, and Microfil were continuously perfused into the left ventricle. The rats were subsequently stored at 4°C overnight. Next day, the skulls were collected, and 10% ethylenediaminetetraacetic acid (EDTA) solution was used for decalcification. Micro‐CT imaging of the skulls was performed at a resolution of 9 µm.

Assessment of bone regeneration: At 12 weeks post‐surgery, the rats were sacrificed, and their skulls were collected. After fixing in formalin, the samples were imaged via micro‐CT at a resolution of 9 µm. The micro‐CT data were analyzed using CTvox, DataViewer, and CTan.

### 5.39 Histological and Immunofluorescence Analyses

In accordance with previous studies [13, 143], skull samples were decalcified with 10% EDTA solution, dehydrated with ethanol, embedded in paraffin, and cut into 5 µm sections. H&E was used to evaluate new bone formation and biosafety. Masson's trichrome staining was used to evaluate new bone formation and collagen deposition. Immunofluorescence staining of CD86 and CD206 was performed to assess macrophage polarization. Immunofluorescence staining of COLI was utilized to evaluate bone regeneration. Immunofluorescence staining of CD31 was performed to assess angiogenesis.

### 5.40 Statistical Analysis

All experiments were performed independently at least three times. Statistical analyses and data visualization were performed using GraphPad Prism 9 (GraphPad Software, San Diego, CA, USA). For RT‐qPCR, relative mRNA expression was calculated using the 2^−ΔΔCt^ method and normalized to the control group, and no collected data points were excluded from the statistical analyses. All data were presented as mean ± standard deviation (SD). One‐way analysis of variance (ANOVA) followed by Tukey's post hoc test was used to compare the means of three or more groups. The sample size (n) for each statistical analysis is indicated in the corresponding figure legend. A *p*‐value < 0.05 was considered statistically significant. Asterisk conventions (*, *p* < 0.05, **, *p* < 0.01, ***, *p* < 0.001, ****, *p* < 0.0001; ns, not significant) and hash conventions (#, *p* < 0.05, ##, *p* < 0.01, ###, *p* < 0.001, ####, *p* < 0.0001) are defined in the figure legends.

## Author Contributions

S.‐C.T., S.‐C.G., J.‐Z.Z., and T.‐H.Z. conceptualized the study. Q.‐S.D., A.W., and P.‐L.L. performed data curation. Q.‐S.D., R.Z., and Z.‐H.S. contributed to formal analysis. S.‐C.T. and S.‐C.G. acquired funding. Q.‐S.D., A.W., and X.‐R.L. performed the investigation. Q.‐S.D., A.W., and S.‐H.H. contributed to methodology. S.‐C.T. and Q.‐S.D. were associated with project administration. F.W., C.‐R.Z., Z.‐Y.H., Z.‐Y.W., and T.‐H.Z. acquired resources. S.‐C.T., S.‐C.G., J.‐Z.Z., and T.‐H.Z. supervised the study. Q.‐S.D., A.W., and J.L. performed validation. Q.‐S.D. performed visualization. Q.‐S.D. wrote the original draft. S.‐C.T. and H.D. reviewed and edited the final manuscript.

## Funding

The present study was supported by the National Natural Science Foundation of China (grant numbers 82072530, 82372566, and 82472595), the Shanghai “Rising Stars of Medical Talent” Youth Development Program (Youth Medical Talents‐Specialist Program), the Shanghai Jiao Tong University School of Medicine “Two‐Hundred Talent” Program (grant number 2022‐017), and the Shanghai Sixth People's Hospital Excellent Young Scientist Development Program (ynyq202101).

## Ethics Statement

Ethical approval was granted by the Ethics Committee of Shanghai Sixth People's Hospital Affiliated to Shanghai Jiao Tong University School of Medicine (approval number: 2021‐0767). All animal experiments were approved by the Animal Care Committee of Shanghai Sixth People's Hospital Affiliated to Shanghai Jiao Tong University School of Medicine.

## Conflicts of Interest

The authors declare no conflicts of interest.

## Supporting information




**Supporting File**: advs78104‐sup‐0001‐SuppMat.docx.

## Data Availability

All information related to this study is available upon reasonable request.
