# Supplementary material for: Yoda1‐Functionalized Nanozymes Coordinate Piezo1 Activation and Mitochondrial Protection to Reprogram the Diabetic Osteoimmune Micromilieu and Promote Vascularized Bone Repair
Source: Adv Sci (Weinh). 2026 Oct 14:e78104. Online ahead of print. doi: 10.1002/advs.78104 (PMC13669222; doi:10.1002/advs.78104)
Supplement: Supplementary file 1 [file ADVS-9999-e78104-s001.docx]

**Supporting Information**

**Figure S1**. TEM image of the MF nanozyme.

**Figure S2**. Bubble chart of representative enriched pathways.

**Figure S3**. (A) Representative immunofluorescence images showing co-localization of MF-FITC@Y (green) with markers for early endosomes (Rab5), mitochondria (TOM20), and lysosomes (LAMP1), illustrating the dynamic intracellular transport pathway of MF@Y nanozymes. (B) Quantitative analysis of Pearson’s correlation coefficients between MF-FITC@Y nanozymes and Rab5, TOM20, or LAMP1. Data are presented as mean ± SD (n = 3).

**Figure S4**. (A) Fluorescence images of DCFH-DA staining of intracellular ROS in HBMSCs and TBHP-stimulated cells after different treatments. (B) Relative quantitative analysis of MitoSOX Green staining. (C) Schematic diagram showing the effects of MF@Y nanozyme on antioxidant and mitochondrial morphology changes. This illustration was created with BioRender.com. (D) Quantitative analysis of JC-10 staining. (E) Relative quantitative analysis of ATP content. (F) Relative quantitative analysis of mPTP integrity. Data are expressed as mean ± SD (n = 3 for B; n = 5 for D, E, F). ****, *p* < 0.0001 (one-way ANOVA followed by Tukey’s post hoc test).

**Figure S5**. (A) Quantitative analysis of p-STING positive cells. (B) Relative mRNA expression levels of *IFNA2*, and SASP factors (*CXCL1* and *CXCL10*). (C) Schematic diagram showing the effects of MF@Y nanozyme on the cGAS‒STING pathway. This illustration was created with BioRender.com. (D) Quantitative analysis of SA-β-gal positive cells. (E) Representative immunofluorescence images of γ-H2AX staining. (F) Relative mRNA expression levels of *p16* and *p21*. (G) Quantitative analysis of EdU-positive cells. Data are expressed as mean ± SD (n = 3 for A, B, F; n = 5 for D, G). **, *p* < 0.01, ***, *p* < 0.001, ****, *p* < 0.0001 (one-way ANOVA followed by Tukey’s post hoc test).

**Figure S6**. (A) Quantitative analysis of migrated cells assessed by Transwell assay. (B) Quantitative analysis of the tube-formation assay. Data are presented as mean ± SD (n = 5 for A; n = 3 for B). ****, *p* < 0.0001 (one-way ANOVA followed by Tukey’s post hoc test).

**Figure S7**. (A) Relative quantitative analysis of ALP staining. (B) Relative quantitative analysis of ARS staining. (C) Relative mRNA expression levels of the osteogenesis-related genes *OCN*, *COL1A1* and *ALP*. (D) Relative quantitative analysis of Oil Red O staining. (E) Relative mRNA expression levels of the adipogenesis-related genes *LPL*, *ADIPOQ, C/EBPα*, and *C/EBPβ*. All data are expressed as the means ± SD (n = 5 for A, B; n = 3 for C, D, E). *, *p* < 0.05, ****, *p* < 0.0001 compared with the TBHP group; ##, *p* < 0.01, ####, *p* < 0.0001 compared with the control group (one-way ANOVA followed by Tukey’s post hoc test).

**Figure S8**. (A) Quantitative analysis of TRAP positive areas. (B) Quantitative analysis of F-actin ring areas. (C) Relative quantitative analysis of bone resorption. Data are presented as mean ± SD (n = 5 for A, B; n = 3 for C). ***, *p* < 0.001, ****, *p* < 0.0001 (one-way ANOVA followed by Tukey’s post hoc test).

**Figure S9**. Quantitative analysis of iNOS-positive and CD206-positive cells. Data are presented as mean ± SD (n = 5). ****, *p* < 0.0001 (one-way ANOVA followed by Tukey’s post hoc test).

**Figure S10**. (A) Micro-CT images of the BCP scaffold. (B) SEM images of the BCP scaffold. (C) Fluorescence images of the pGelMA hydrogels. (D) SEM images of the pGelMA hydrogels. (E) Live/Dead staining of HBMSCs after 3D culture on different hydrogels for 5 days and 7 days. (F) Mass loss of BCPG-MF@Y scaffolds during *in vitro* degradation. Data are presented as mean ± SD (n = 3). (G) Cumulative release profiles of MF nanozyme and Yoda1 from BCPG-MF@Y scaffolds during *in vitro* degradation. Data are presented as mean ± SD (n = 3). (H) Zeta potential of supernatants collected from BCPG-MF@Y scaffolds at successive time points during the *in vitro* degradation process. Data are presented as mean ± SD (n = 3). (I) Fluorescence images of F-actin-stained HBMSCs after culture on different scaffolds.

**Figure S11**. (A) Bioluminescence imaging of the *in vivo* biodistribution of Cy7-labeled pGelMA within BCPG-MF@Y scaffolds implanted in calvarial defects of diabetic rats. (B) Quantitative analysis of bone volume, BV/TV, and trabecular number derived from micro-CT images at 12 weeks post-implantation. Quantitative analysis of H&E (C) and Masson’s trichrome (D) staining reflecting new bone formation and collagen deposition, respectively. (E) Representative immunofluorescence images of CD31 staining. Relative quantitative analysis of CD86 (F) and CD206 (G) staining. Data are presented as mean ± SD (n = 3). **, *p* < 0.01, ***, *p* < 0.001, ****, *p* < 0.0001 (one-way ANOVA followed by Tukey’s post hoc test).

**Figure S12**. H&E-stained sections of heart, liver, spleen, lung, and kidney at week 12 after surgery.

**Table S1.** pH values of the supernatant during degradation of the BCPG-MF@Y scaffold at different time points (mean ± SD, n = 3).

| Time (days) | pH |
| --- | --- |
| 3 | 7.142 ± 0.069 |
| 5 | 7.259 ± 0.040 |
| 7 | 7.266 ± 0.057 |
| 14 | 7.018 ± 0.061 |
| 21 | 7.076 ± 0.065 |
| 28 | 7.168 ± 0.031 |
| 35 | 7.180 ± 0.070 |
| 42 | 7.186 ± 0.090 |

**Table S2.** Sequences of the primers used for RT-qPCR.

| Gene | Forward Primer Sequence (5’-3’) | Reverse Primer Sequence (5’-3’) |
| --- | --- | --- |
| *p16* | GAT TGA AAG AAC CAG AGA GGC T | GAC CTT CGG TGA CTG ATG AT |
| *p21* | CAC TTT GAT TAG CAG CGG AAC | CAA CTA CTC CCA GCC CCA TAT |
| *MMP1* | GTC AAG TTT GTG GCT TAT GGA | TTG TCA CTG AAG CTG CTC TCT |
| *IL-6* | AAC AAC CTG AAC CTT CCA AAG | CAA ACT CCA AAA GAC CAG TGA |
| *CXCL1* | AAC ATG CCA GCC ACT GTG ATA | ACA TAC ATT CCC CTG CCT TCA |
| *IFNA1* | TAC ACC AGG TCA CGC TTT C | ATC AGT TTA TCA GCA TGG TCA TAG |
| *IFNB1* | GGT TAC CTC CGA AAC TGA AGA | GCT GGT TGA AGA ATG CTT G |
| *COL1A1* | TAG TCT GTC CTG CGT CCT CTG | GAG TCT TTT GCT TCC TCC CAC |
| *OCN* | CCA GGA GGG AGG TGT GTG | TGA AAG CCG ATG TGG TCA |
| *ALP* | ACC ACC ACG AGA GTG AAC CA | CGT TGT CTG AGT ACC AGT CCC |
| *ANGPT1* | GCT TGG TTA CTC GTC AAA CAT | GCT TCT GAA GGA CAC TGT TGT |
| *PDGFB* | GCT TCT TTT CGT TTT CGT TTT G | ACT CCA TCT CTA ACC CAC CTG A |
| *AGGF1* | TCT AAA GTC TCA GGG TTC CAC | TTA CAG ATG GAA ATA GAC CCA G |
| *TEK* | GCC ACC ATC ACT CAG TAT CAG | GTT GCT TGA CCC TAT GTT GTT C |
| *AAMP* | GGC TAC CTG GAT GGG ACC TT | TGG TGC TGA CAC TGA TGC CTA |
| *LPL* | TCA TTC CCG GAG TAG CAG AGT | GGC CAC AAG TTT TGG CAC C |
| *C/EBPα* | AGG AGG ATG AAG CCA AGC AGC T | AGT GCG CGA TCT GGA ACT GCA G |
| *C/EBPβ* | AGA AGA CCG TGG ACA AGC ACA G | CTC CAG GAC CTT GTG CTG CGT |
| *ADIPOQ* | GCT ATT TTG TGC CCA CTG T | TAA CTC TCT CAG CCT CAG GAT |
| *β-actin* | CCT CTA TGC CAA CAC AGT | AGC CAC CAA TCC ACA CAG |
